# A single active catalytic site is sufficient to promote transport in P-glycoprotein

**DOI:** 10.1038/srep24810

**Published:** 2016-04-27

**Authors:** Orsolya Bársony, Gábor Szalóki, Dóra Türk, Szabolcs Tarapcsák, Zsuzsanna Gutay-Tóth, Zsolt Bacsó, Imre J. Holb, Lóránt Székvölgyi, Gábor Szabó, László Csanády, Gergely Szakács, Katalin Goda

**Affiliations:** 1 Department of Biophysics and Cell Biology, University of Debrecen, P.O. Box 400, Debrecen H-4002, Hungary; 2 Institute of Enzymology, Research Centre for Natural Sciences, Hungarian Academy of Sciences, Hungary; 3 Institute of Horticulture, University of Debrecen, P.O. Box 36, H-4015 Debrecen, Hungary; 4 Plant Protection Institute, Centre for Agricultural Research, Hungarian Academy of Sciences, H-1525 Budapest, Hungary; 5 MTA-DE Momentum, Genome Architecture and Recombination Research Group, Department of Biochemistry and Molecular Biology, University of Debrecen, Debrecen H-4032, Hungary; 6 MTA-SE Ion Channel Research Group, Budapest H-1094, Hungary; 7 Institute of Cancer Research, Department of Medicine I, Comprehensive Cancer Center, Medical University of Vienna, Vienna, Austria

## Abstract

P-glycoprotein (Pgp) is an ABC transporter responsible for the ATP-dependent efflux of chemotherapeutic compounds from multidrug resistant cancer cells. Better understanding of the molecular mechanism of Pgp-mediated transport could promote rational drug design to circumvent multidrug resistance. By measuring drug binding affinity and reactivity to a conformation-sensitive antibody we show here that nucleotide binding drives Pgp from a high to a low substrate-affinity state and this switch coincides with the flip from the inward- to the outward-facing conformation. Furthermore, the outward-facing conformation survives ATP hydrolysis: the post-hydrolytic complex is stabilized by vanadate, and the slow recovery from this state requires two functional catalytic sites. The catalytically inactive double Walker A mutant is stabilized in a high substrate affinity inward-open conformation, but mutants with one intact catalytic center preserve their ability to hydrolyze ATP and to promote drug transport, suggesting that the two catalytic sites are randomly recruited for ATP hydrolysis.

P-glycoprotein (Pgp) is a primary active membrane transporter of the ABC (ATP Binding Cassette) protein superfamily. It is the first human ABC transporter discovered to be responsible for the increased efflux of chemotherapeutics from multidrug resistant cancer cells^1^,^2^. Pgp is referred to as a “hydrophobic vacuum cleaner”, because it is believed to extract its substrates directly from the inner leaflet of the plasma membrane^3^. This molecular mechanism of action provides an incredibly efficient efflux of a vast array of hydrophobic drugs, ensuring the survival of cancer cells despite toxic chemotherapy (for reviews see^4^,^5^).

Based on biochemical experiments and the commonality of various mammalian and bacterial ABC transporter structures it is generally believed that the fundamental molecular mechanism of substrate transport is shared among ABC transporters^6^,^7^,^8^. From bacteria to humans, ABC transporters are composed of at least two membrane-embedded transmembrane domains (TMDs) and two cytoplasmic nucleotide binding domains (NBDs). The TMDs define the substrate binding sites and the translocation pathway, and the NBDs bind and hydrolyze ATP. The TMDs are connected to the NBDs by intra-cytosolic loop (ICL) domains, which transfer signals to coordinate ATP binding and hydrolysis with substrate transport^7^. The NBDs contain several evolutionarily conserved sequences, including the Walker A and B motifs that are commonly found in nucleotide-binding proteins^9^, and the so-called signature sequence (C-loop or LSGGQ motif), which is unique to the ABC protein family^10^. The conserved motifs form two composite catalytic sites in which the ATP molecules are sandwiched between the Walker A and Walker B motifs of one NBD and the signature sequence of the contralateral NBD^11^.

Crystal structures of full-length ABC transporters have revealed two major conformations: in the absence of nucleotides, the NBD dimers are dissociated and the TMDs adopt an inward-facing conformation^6^,^12^. Nucleotide binding to the dissociated NBDs^13^ induces formation of two composite catalytic sites at the interface of a tight head-to-tail NBD1/NBD2 heterodimer, glued together by the two ATP molecules. Concomitantly with NBD dimer formation the TMDs flip into an outward-facing conformation^7^,^8^. Only one of two gates is open at any time: in the inward-facing conformation the cytoplasmic gate of the translocation pathway is open, whereas the extracellular gate is closed. Conversely, in the outward-facing conformation the cytoplasmic gate is closed and the transported drug is free to dissociate to the extracellular compartment. The above conformational transitions are accompanied by the switch of the affinity of the substrate binding sites from high- to low-affinity to ensure substrate transport against the concentration gradient^14^,^15^.

Notably, the recently solved structure of the antibacterial peptide ABC transporter McjD is occluded on both sides of the membrane. This conformation, termed nucleotide-bound outward occluded, probably represents a transition intermediate between the outward-open and inward-open TMD conformations of ABC exporters^16^. Repeated formation and disruption of the NBD dimer is generally agreed to involve the cooperative, ATP-dependent interaction of the NBDs, but because the resolved structures do not represent all phases of the transport cycle the exact sequence of events is unknown. While the major principles of the ATP-dependent transport mechanism are shared among ABC transporters, details of the coupling of the TMD transitions to the ATPase cycle may differ in different subclasses of ABC exporters^17^.

A spate of biochemical data supports that the molecular mechanism of Pgp follows the above alternating access scheme (reviewed in^18^,^19^). Still, despite the availability of the mouse^6^,^20^ and the *C. elegans*^12^ Pgp structures, the molecular mechanisms linking ATP binding and/or hydrolysis to the association and dissociation of NBDs and to conformational changes occurring in the TMDs are not fully understood. The catalytic cycle is driven by ATP hydrolysis, which may happen at either of the two functionally symmetrical composite nucleotide binding sites. How ATP hydrolysis is coordinated between the two NBDs, and whether Pgp hydrolyses one or two (or more) ATP molecules per each transported substrate are not known. Experiments attempting to explain the sequence of molecular events that lead to the ATP-dependent conformational switch and the change of substrate binding affinity have produced competing models^21^.

Conformational transitions in Pgp that occur during transport-associated ATP hydrolysis can be detected by differential immunoreactivity to the monoclonal antibody UIC2^22^. The complex epitope of UIC2 consists of multiple extracellular loops of Pgp^23^,^24^, which undergo conformational changes when the TMDs rearrange to bind and release the transported substrate. It was shown that UIC2 binding to Pgp is increased in the presence of transported substrates, ATP-depleting agents, or by mutational inactivation of both nucleotide-binding domains^22^, suggesting that UIC2-detectable conformational transitions are driven by binding and unbinding of nucleotides^25^. In the present study we correlated UIC2-reactivity with drug binding affinity for wild-type and mutant Pgp variants in permeabilized cells to elucidate the link between ATP binding, hydrolysis and the conformational rearrangements responsible for switching the affinity of the substrate binding sites during substrate transport.

## Results

### Nucleotide binding switches Pgp into the outward facing conformation

**Figure 1:**
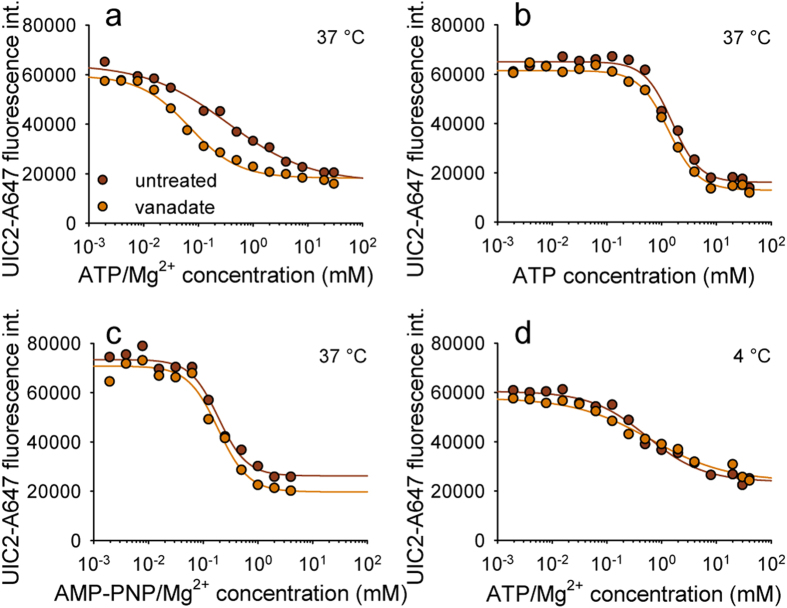
UIC2 mAb binding to Staphylococcus alpha toxin permeabilized NIH 3T3 cells expressing wild-type Pgp in the presence of ATP/Mg^2+^ (**a**) ATP in the absence of Mg^2+^ (**b**) AMP-PNP/Mg^2+^ (**c**) or ATP/Mg^2+^ at 4 °C (**d**). Permeabilized cells were treated with nucleotides at different concentrations for 20 min and then labeled with UIC2-A647 for another 30 min (**a–c**) or 40 min (**d**) without washing out the nucleotides. In case of V_i_ treatment un-trapped nucleotides were washed out in the presence of V_i_. UIC2-A647 fluorescence intensities were plotted as a function of nucleotide concentration and fitted with a four parameter Hill function to determine the apparent affinities of nucleotide binding (*K*_*A*_) and Hill slope values (see Materials and Methods).

Gentle permeabilization of cells allows selective modulation of the intracellular milieu while preserving membrane integrity and the function of transmembrane transporters. This experimental setup has been used before to synchronize Pgp molecules in an ATP-free, high UIC2-affinity conformation^25^. Similarly to the results obtained by Druley and co-workers^25^, replenishing the cells with ATP/Mg^2+^ shifted Pgp into a low UIC2-affinity conformation in a concentration dependent manner (Fig. 1a). The apparent affinity of ATP for competing UIC2 labeling (K_A_, Supplementary Table S1) was found to be comparable with reported K_M_ values for ATP hydrolysis^26^,^27^ as well as with our results obtained in membrane samples prepared from Pgp^+^ NIH 3T3 cells (Supplementary Fig. S1).

AMP-PNP, a non-hydrolysable ATP analogue also induced a conformation change in the transmembrane domains resulting in a concentration dependent decrease in UIC2 reactivity (Fig. 1c). When ATP was added in the absence of Mg^2+^ (ATP + EDTA; Fig. 1b) or on ice (Fig. 1d), i.e., under conditions that prevent ATP hydrolysis, the conformational change driving Pgp into a UIC2-dim state was observed at significantly higher nucleotide concentrations (Supplementary Table S1).

The transport and ATPase cycle of Pgp is inhibited by phosphate-mimicking anions, such as orthovanadate (V_i_), which can block the protein by stably replacing the cleaved gamma phosphate. The complex consisting of Pgp, ADP and vanadate (Pgp-ADP-V_i_) is formed only under conditions that allow hydrolysis of at least one ATP molecule, and is generally accepted to closely mimic the conformation of a transition state^28^. Of note, consistent with the formation of the Pgp-ADP-V_i_ complex, addition of vanadate increased the apparent affinity under hydrolysis conditions (37 °C, presence of ATP/Mg^2+^, Fig. 1a, Supplementary Table S1). However, when ATP hydrolysis was prevented, the apparent affinity was not influenced at all by V_i_ (Fig. 1b–d), confirming that ATP hydrolysis and the subsequent release of the *γ*-phosphate is a prerequisite for the formation of the Pgp-ADP-V_i_ complex^29^.

### Unilateral mutation of the Walker A lysine residues allows ATP binding and substrate-stimulated ATP hydrolysis

**Figure 2:**
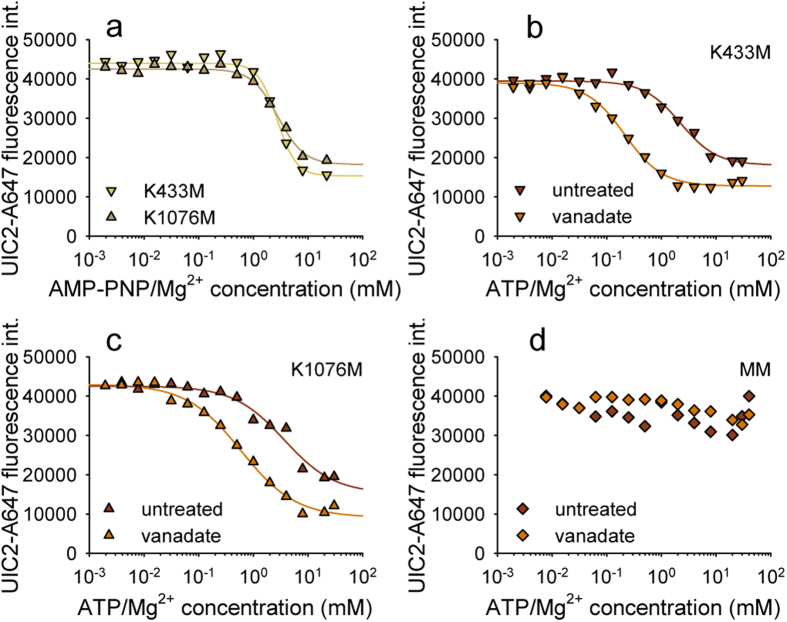
UIC2 mAb binding to single or double Walker A mutant Pgp variants in permeabilized cells in the presence of AMP-PNP/Mg^2+^ (**a**) or ATP/Mg^2+^ (**b–d**) in the absence or presence of V_i_. Permeabilized cells were treated with nucleotides at different concentrations for 20 min and then labeled with UIC2-A647 for another 30 min without washing out the nucleotides. In case of V_i_ treatment un-trapped nucleotides were washed out in the presence of V_i_. UIC2-A647 fluorescence intensities were plotted as a function of nucleotide concentration and fitted with a four parameter Hill function to determine the apparent affinities of nucleotide binding (*K*_*A*_) and Hill slope values (see Materials and Methods).

The Walker A motif (GXXGXGKS/T), also known as the P-loop (phosphate-binding loop), forms extensive bonds with the terminal phosphates of the bound nucleotide^9^. Mutation of the highly conserved lysine residue to methionine was shown to abolish ATPase activity of several ABC transporters^30^,^31^,^32^. To study the effect of mutations of the Walker A lysines in NBD1 (K433) and NBD2 (K1076) of Pgp on nucleotide-induced TMD conformational flexibility, we engineered mammalian cell lines (NIH 3T3 and MDCKII) stably expressing wild-type (WT), K433M, K1076M, or K433M/K1076M P-glycoprotein using a *Sleeping Beauty* transposon-based gene delivery system. Consistent with previous studies that showed that single Walker A mutations allow nucleotide binding^32^, UIC2-reactivity of the K433M and K1076M variants decreased in the presence of AMP-PNP and ATP (Fig. 2a–c). Earlier we have shown the fluoroaluminate-dependent labeling of K433M and K1076M Pgp with [α-^32^P]-8-azido-ATP^33^. Here we find that addition of vanadate results in a 5–10-fold increase in the apparent affinity of the single mutants to ATP (Fig. 2b,c). Because complex formation with vanadate requires prior hydrolysis of ATP, these effects of vanadate indicate that Pgp is capable of ATP hydrolysis despite the mutation of a single Walker A lysine residue. In contrast, simultaneous mutation of both lysine residues resulted in a stable UIC2-binding conformation, which could not be reverted by nucleotides (Fig. 2d).

**Figure 3:**
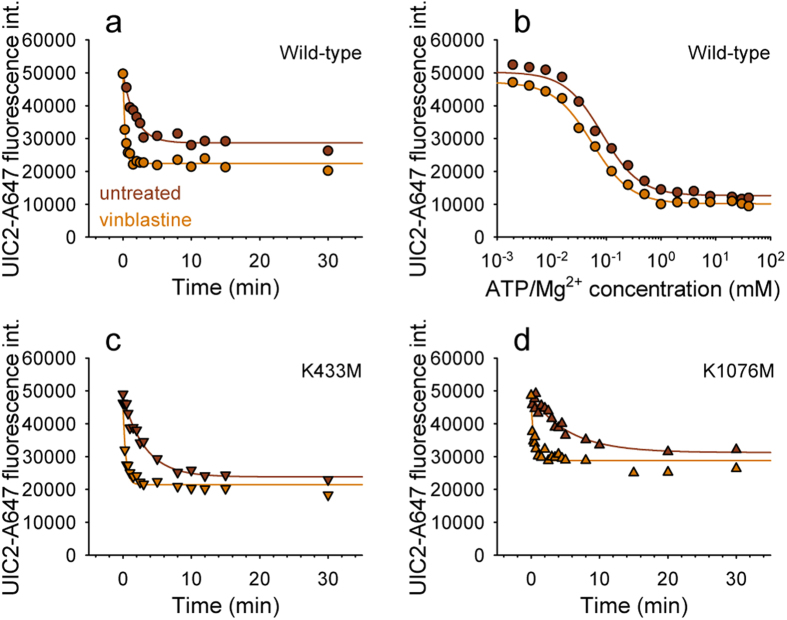
Kinetics of the formation of the BeF_x_-trapped species in case of wild-type (**a**) and single mutant (**c,d**) Pgp expressing cells. Permeabilized cells were incubated with 100 μM (for wild-type) or 500 μM (for K433M and K1076M) ATP/Mg^2+^ and BeF_x_ (200 μM BeSO_4_ and 1 mM NaF) in the presence or absence of 50 μM vinblastine at 37 °C and samples were taken at different time points. Panel (**b**) shows the effect of 200 μM vinblastine on the apparent nucleotide affinity of wild-type Pgp. Permeabilized cells were treated with BeF_x_ and different concentrations of ATP/Mg^2+^ in the presence or absence of 200 μM vinblastine for 20 min at 37 °C. UIC2-A647 labeling was carried out following the washout of ATP/Mg^2^ in the maintained presence of BeF_x_.

The turnover rate of Pgp’s catalytic cycle is accelerated in the presence of transported substrates (substrate-stimulated ATPase activity)^27^. Kinetic analysis of the formation of the BeF_x_- or V_i_-trapped complexes showed that progressive accumulation in the low-UIC2-affinity trapped transition state occurs at comparable rates in WT or single mutant Pgps. Accumulation in the trapped complex was accelerated by Pgp substrates (e.g. verapamil, vinblastine, rhodamine 123) or competitive inhibitors (e.g. cyclosporine A) both in WT and single Walker A mutant Pgp variants (results are shown for vinblastine, see Fig. 3a,c,d), while the apparent affinity for ATP did not change even at high substrate concentrations (up to 200 μM vinblastine, see Fig. 3b). The effect of transported substrates on the rate of formation of the trapped transition state complex was identical in the WT and the single mutant Pgp variants (Supplementary Table S3). These data suggest that despite the mutation of a single critical Walker A lysine residue, the catalytic cycle can progress to the step arrested by phosphate mimicking anions. The relatively long t_1/2_ values of BeF_x_ (~120 s, see Fig. 3 and Supplementary Table S3) and V_i_ trapping (*t*_*1/2*_ = 235.2 ± 52.2 s; n = 6) compared to the total cycle time which is on the order of 100 ms suggest that formation of a stable post-hydrolysis complex by phosphate mimicking anions is an extremely low-probability event, likely reflecting the very short time window of the vanadate- or BeF_x_-“sensitive” state in each cycle, that is between dissociation of the cleaved phosphate and disassembly of the NBD dimer. Thus, similarly to WT Pgp, single Walker A mutant Pgp variants can not only hydrolyze ATP, but pass on average ~1200–2500 cycles before trapping by BeF_x_ or vanadate occurs. Consistently with this turnover, single mutants retain a weak, but significant verapamil stimulated ATPase activity that can be detected in membrane samples prepared from NIH 3T3 cells expressing the transporter at high levels (Supplementary Fig. S2).

### The rate of dissociation of the V_i_-trapped complex is decreased by single Walker A mutations

**Figure 4:**
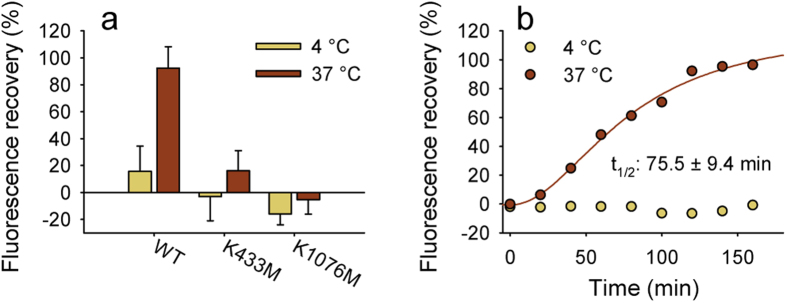
Recovery of UIC2 labeling of wild-type and single Walker A mutant Pgp. (**a**) Staphylococcus aureus alpha toxin-permeabilized NIH 3T3 cells expressing wild-type or single Walker A mutant Pgp were incubated with ATP and 500 μM vanadate (V_i_) to induce the formation of trapped Pgp-species. UIC2 mAb labeling was carried out after a 120-minute incubation at the indicated temperatures in V_i_-free solution containing 50 μM NEM in the absence of ATP. (**b**) Time-dependent recovery of the UIC2-binding conformation of wild-type Pgp. Aliquots washed for various time intervals in V_i_- and ATP-free solution containing 200 μM NEM were subsequently labeled with UIC2 mAb; washing and labeling were carried out either at 4 °C or at 37 °C.

Although the V_i_-trapped species represent a dead-end of the catalytic reaction cycle, we wondered if the eventual release of the trapped nucleotide could be detected by an increase of UIC2 reactivity. Following the removal of uncomplexed V_i_, complete time-dependent recovery of the UIC2-reactive state corresponding to the release of nucleotides was indeed observed (Fig. 4b). The half-life of Pgp-ADP-V_i_ complexes was 75.1 ± 5.9 min, in good agreement with literature data^34^,^35^. Release of the tightly bound nucleotide was temperature dependent (Fig. 4a,b), but was not influenced by the presence of ATP and transported substrates such as vinblastine or verapamil (not shown). UIC2 binding to the mutant Pgp variants was not restored in the time frame of the experiment (up to 4 hours), suggesting that mutation of a single Walker A residue increases the life time of the vanadate-trapped complex.

### Single Walker A mutants are transport-competent

**Figure 5:**
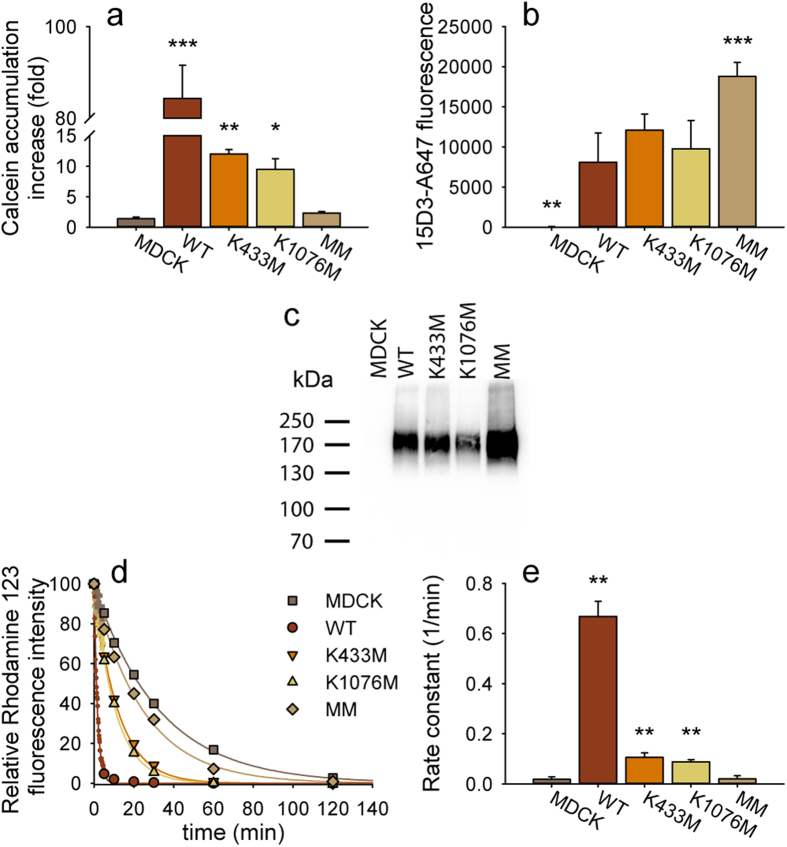
Functional expression of wild-type and Walker A mutant human Pgp variants in MDCK cells. (**a**) Cells were incubated with 0.1 μM calcein-AM with or without the Pgp-inhibitor cyclosporin A (CsA, 10 μM) for 30 minutes. Bars show the relative increase of calcein accumulation caused by CsA (means of 3 independent experiments ± SD). (**b**) Mean fluorescence of Pgp-expressing cells stained with the anti-Pgp 15D3-A647 monoclonal antibody, showing comparable expression of Pgp variants in MDCK cells. *P < 0.05, **P < 0.01, ***P < 0.001. In panel (**a)** samples were compared to the Pgp non expressing MDCK cells, while in panel (**b**) the samples were compared to the wild-type Pgp (WT) expressing cells. (**c**) Western blot analysis of Pgp expression using a monoclonal anti-Pgp mAb (G-1, Santa Cruz Biotechnology Inc.). (**d**) Time dependent efflux of R123 from preloaded cells. Preloaded cells were incubated at 37 °C; during the first 5 min the fluorescence intensity of the cells was measured continuously and the values were averaged for 10 s intervals. At later time points fluorescence intensity distribution histograms were measured and their median fluorescence intensities were determined. (**e**) First order rate constants of the exponential curves fitted to the R123 efflux data (mean ± SD of three independent experiments). Significant differences compared to the non-transfected MDCK cells are shown by **P < 0.01.

Because single Walker A mutants were found to exhibit substrate-stimulated ATP hydrolytic activity (Fig. 3 and Supplementary Fig. S2), we next set out to determine whether they are also capable of promoting substrate transport. We compared the accumulation of calcein in both NIH 3T3 and MDCK cells expressing WT or mutant Pgp. Despite comparable expression levels, the Pgp-variant missing both Walker A lysine moieties (MM) was unable to hinder accumulation of calcein in the cells, whereas cells transfected with the single mutants showed a cyclosporine A-sensitive reduction in calcein fluorescence (Fig. 5a. and Supplementary Fig. S3). Reduction of intracellular calcein levels is a result of uphill transport activity which, by thermodynamic arguments, must be driven by ATP hydrolysis. To provide a quantitative measure of this residual transport activity, we determined the first order rate constants (*k*) of rhodamine123 (R123) efflux^36^ from cells expressing wild-type or mutant Pgp at comparable levels (Fig. 5b,c). In agreement with the data obtained with the calcein assay, single mutants showed significant R123 efflux activity, while the double mutant was inactive (Fig. 5d,e). In line with the relatively weak transport activity, single mutants conferred resistance against the cytotoxic effect of vinblastine (Supplementary Fig. S4). Collectively, these results suggest that single Walker A mutants retain a residual transport activity of ~15% of WT, whereas the double mutant is not transport competent.

### Mutation of the critical Walker A lysines results in increased drug binding affinity

**Figure 6:**
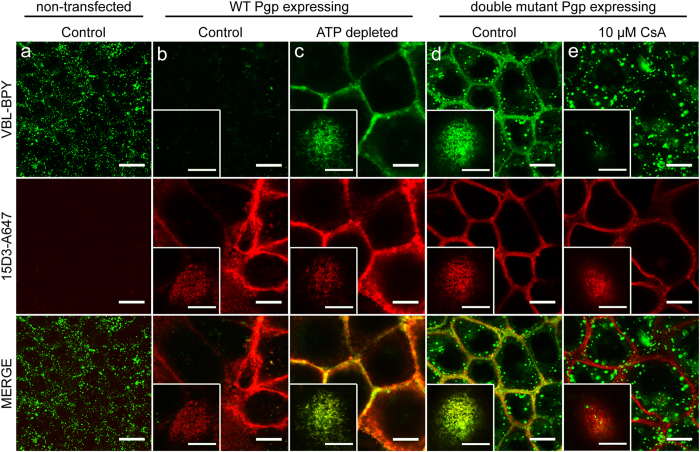
Accumulation of Bodipy FL Vinblastine (VBL-BPY) in the apical plasma membrane of energy deprived wild-type (WT) Pgp expressing and double Walker A mutant (K431M/K1076M) Pgp expressing MDCK cells reveals the stabilization of a high-affinity substrate binding Pgp conformation. VBL-BPY (green) and anti-Pgp 15D3-Alexa647 mAb (15D3-A647; red) staining of confluent non-transfected MDCK cells (**a**) wild-type Pgp-expressing (**b,c**) and Walker A mutant (K431M/K1076M) Pgp expressing MDCK cells (**d,e**). ATP depletion was induced by 30 min pre-treatment with 10 mM Na-azide and 8 mM 2-deoxyglucose treatment (**c**). A non-fluorescent competitive Pgp inhibitor CsA (10 μM) was added 10 min before VBL-BPY and antibody staining (**e**). Inserts show the apical membrane surface of one cell. Bar: 10 μm.

Consistently with the Pgp-mediated efflux of vinblastine-bodipy (VBL-BPY), cells expressing WT Pgp show significantly lower levels of intracellular fluorescence as compared to Pgp^−^ cells when incubated with this fluorescent drug (Fig. 6, b vs. a). ATP-depleted cells overexpressing wild-type Pgp sequester VBL-BPY in the plasma membrane (Fig. 6c). Strikingly, plasma membrane sequestration of VBL-BPY was also observed in cells transfected with either single or double Walker A mutant Pgp variants (for double Walker A mutant see Fig. 6d). Addition of cyclosporine A (CsA), a competitive inhibitor of Pgp, prevented plasma membrane accumulation of VBL-BPY (Fig. 6e), suggesting that the enrichment of VBL-BPY in the plasma membrane is in each case due to its high-affinity binding to Pgp.

**Figure 7:**
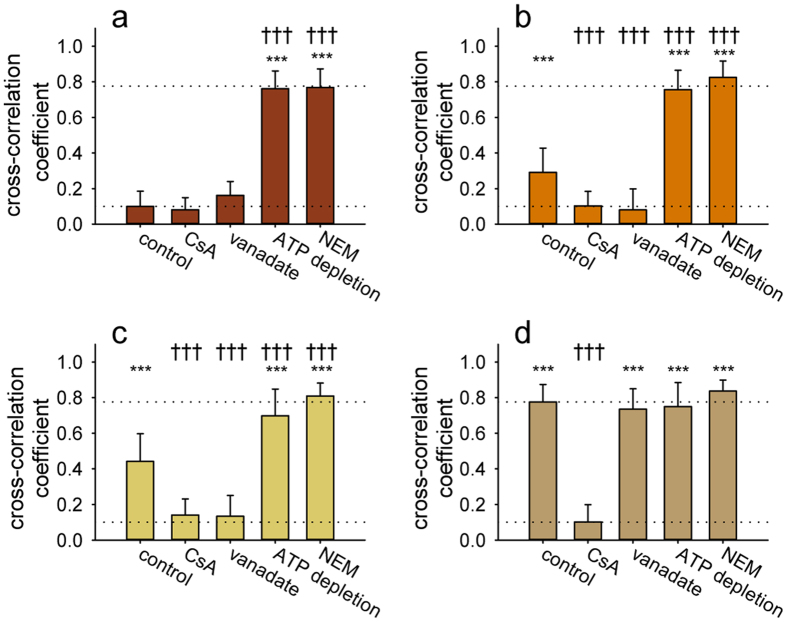
Pearson’s cross-correlation coefficients between the intensity distributions of cell-surface BODIPY FL Vinblastine (VBL-BPY) and 15D3-Alexa-647 labeling calculated for wild-type (**a**) single K433M (**b**); K1076M (**c**) and double Walker A mutant Pgp variants (**d**) expressed in MDCK cells. Cells were pre-treated for 30 min at 37 °C with 10 μM cyclosporine A (CsA), 2 mM vanadate (V_i_), 50 μM N-ethyl-maleimide (NEM), or 10 mM Na-azide and 8 mM 2-deoxy D-glucose (ATP depletion), and then further incubated with VBL-BPY and 15D3-A647 anti-Pgp antibody for another 30 min at 37 °C. Significant differences compared to the wild-type’s untreated control are shown by ***P < 0.001, **P < 0.01, or *P < 0.05 and those compared to the untreated control of the respective cell line by: ^†††^P < 0.001, ^††^P < 0.01, or ^†^P < 0.05. The untreated controls of the single and double mutants were also significantly different from each other (P < 0.01).

To quantify the fraction of substrate-bound Pgp molecules in the membrane, Pearson’s cross-correlation coefficients were calculated between the VBL-BPY and 15D3 anti-Pgp antibody signals in single optical slices of apical membrane surfaces. Since the antibody staining was unchanged in the course of the treatments, the cross-correlation coefficients depend mostly on VBL-BPY binding to Pgp. In cells expressing double Walker A mutant Pgp (Fig. 7d) large cross-correlation coefficients of close to unity (~0.8) indicate that the majority of Pgp molecules are in high substrate affinity conformation. Single Walker A mutants also exhibited somewhat increased drug binding compared to the WT (Fig. 7b,c), but this binding was fully suppressed by transition state analogs, e.g. vanadate (Fig. 7b,c). Furthermore, ATP depletion by Na-azide or prevention of ATP binding by NEM treatment^29^ shifted both single mutant and WT Pgp variants into the substrate binding conformation, as reflected by increased co-localization of VBL-BPY and Pgp molecules in the plasma membrane (Fig. 7a–c). Remarkably, the high substrate affinity of double mutant Pgp was not affected by any of the above treatments (Fig. 7d).

### AMP-PNP binding is sufficient to switch Pgp from high to low drug affinity conformation

**Figure 8:**
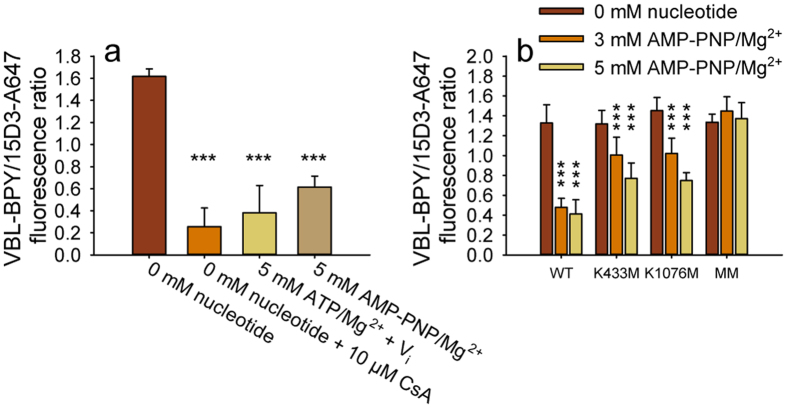
BODIPY FL Vinblastine (VBL-BPY) binding to wild-type Pgp in permeabilized NIH 3T3 cells (**a**) and to Walker A mutant Pgps in permeabilized MDCK cells (**b**). Cells permeabilized by Staphylococcus alpha toxin (**a**) or Streptolysin O (**b**) were treated with different concentrations of CsA, ATP/Mg^2++^ vanadate (V_i_) or AMP-PNP/Mg^2+^ followed by co-staining with VBL-BPY and 15D3-A647. The ratio of the VBL-BPY and 15D3-A647 fluorescence intensity was determined in pixels representing the plasma membrane, selected on the basis of 15D3-A647 fluorescence intensity exceeding the threshold of 300. Nucleotide-treated samples were compared to permeabilized ATP-depleted cells (0 mM nucleotide); the values are means ± SD of three independent experiments (***P < 0.001).

In permeabilized cells ATP depletion synchronized WT Pgp molecules in a high drug binding affinity conformation, as indicated by VBL-BPY sequestration in the plasma membrane (Fig. 8a, brown bar). Suppression of membrane fluorescence by CsA confirmed that drug accumulation in the membrane was indeed due to tight binding of VBL-BPY to Pgp (Fig. 8a, orange bar). This tight binding of VBL-BPY to Pgp was reversed in the presence of ATP and vanadate (Fig. 8a, ocher bar), consistent with the low drug binding affinity of the transition state complex^37^. Strikingly, incubation of permeabilized cells in 5 mM AMP-PNP also resulted in a significant decrease in the co-localization of VBL-BPY and Pgp, indicating that ATP hydrolysis is not required for the affinity switch responsible for the release of the transported substrate (Fig. 8a, last bar). AMP-PNP treatment resulted in a similar, although smaller, reduction of VBL-BPY binding to the single mutants (Fig. 8b). In contrast, simultaneous mutation of both Walker A lysine residues resulted in a permanent high drug binding affinity conformation, which could not be reversed by the addition of nucleotides (Fig. 8b).

## Discussion

Based on crystal structures and a wealth of biochemical and biophysical data it is generally accepted that ABC transporters adopt at least two discrete conformations along their transport cycle. The switch between the NBD-dissociated, inward-facing and the NBD-associated, outward-facing states involves a series of conformational changes which ultimately result in the reduction of substrate binding affinity required for uphill substrate transport. The exact molecular mechanisms that link nucleotide binding to the association and dissociation of NBDs and to the conformational changes of the TMDs are not fully understood. Our aim was to capitalize on the unique property of UIC2, a conformation-sensitive monoclonal antibody which recognizes a complex extracellular Pgp epitope, to follow the conformational changes which occur in the TMDs. UIC2 distinguishes two conformations of Pgp, which can be studied by modulating intracellular ATP-levels in *Staphylococcus aureus* alpha-toxin-permeabilized cells overexpressing Pgp^25^: the UIC2-dim and UIC2-reactive conformations correspond to the outward- and inward facing conformations observed in ATP-bound and nucleotide-free crystal structures, respectively. In addition, we monitored the nucleotide-dependent switch in drug binding affinity using a fluorescent Pgp substrate analog. Together, these assays allowed us to elucidate the link between ATP binding, hydrolysis and the conformational rearrangements responsible for switching the affinity of the substrate binding sites during substrate transport.

The timing of the conformational change which results in the decrease of drug binding affinity has been a matter of much debate. The “ATP switch model” predicts that ATP binding induces dimerization of the NBDs, which then triggers conformational changes resulting in the decrease of drug binding affinity; whereas the dissociation of the NBD resets the transporter for the next cycle^38^,^39^,^40^,^41^,^42^. In accordance with the above model the AMP-PNP-bound state of Pgp was reported to show reduced binding of vinblastine^43^,^44^. In some studies transition from the high to the low substrate affinity state was found to be triggered by nucleotide occlusion (i.e. tight binding) within the formed NBD dimer^42^,^45^,^46^. In contrast, other studies found that the conformational switch coupled to the change in drug binding affinity is driven by ATP hydrolysis^47^,^48^,^49^ or by the relaxation of a high-energy catalytic site conformation generated by the hydrolysis step^28^. These conflicting data may be due to the inherent limitations of the different experimental approaches such as the low labeling stoichiometry and the variable labeling efficiency of photoaffinity reagents, as well as the difficulty of quantifying binding affinity of highly lipophilic compounds to transmembrane transporters^50^,^51^.

In view of the intimate association of Pgp with the lipid bilayer in which it is embedded, and from which it harvests its substrates, it is worth noting that the confocal microscopic fluorescence co-localization assay and the UIC2 assay shown here characterize the transporter in its natural plasma membrane environment. Although the UIC2 assay is an indirect method for measuring the apparent affinities of nucleotides to Pgp, our experimental system is clearly capable of discriminating between distinct steps of the ATPase cycle such as nucleotide binding or ATP hydrolysis. Our results demonstrate that AMP-PNP binding is sufficient to induce the conformational switch corresponding to the transition from the inward to the outward facing conformation (Fig. 1c). Using the same experimental setup, we also show that binding of AMP-PNP switches Pgp into the low drug binding affinity state (Fig. 8a). The simultaneous drop in the UIC2- and drug-binding affinities suggests that the high-to-low switch in drug binding affinity might coincide with the transition from the inward- to the outward-facing conformation, but in any case precedes ATP hydrolysis (Figs 1,2 and 8.).

Residues of the Walker motifs in each NBD, together with the signature sequence of the contralateral NBD, directly participate in nucleotide-dependent dimerization of the two NBDs and ATP hydrolysis. How ATP hydrolysis is coordinated between the two NBDs, and whether Pgp hydrolyses one or two (or more) ATP molecules per each transported substrate are not known. In diverse ABC transporters, mutations of the conserved Walker A lysine reduce ATPase activity to very low levels^30^,^31^,^32^,^52^,^53^,^54^. The two nucleotide binding domains of Pgp were shown to be functionally equivalent and the integrity of both catalytic centers is generally believed to be needed for transport, because inactivation of a single NBD results in inhibition of ATPase and transport activities^29^,^34^,^55^.

We confirm that mutation of both Walker A lysine residues inactivates Pgp (Fig. 5 and Supplementary Fig. S3): as indicated by the lack of ATP-triggered conformational changes, the transporter is essentially frozen in the UIC2-reactive inward-open state (Fig. 2) characterized with high drug binding affinity (Figs 6, 7, 8). Intriguingly, however, our data show that nucleotide binding to single Walker A mutants triggers the same inward-to-outward conformational switch (Fig. 2a), and the concomitant drop in drug binding affinity (Figs 7 and 8), as observed for the WT protein. Furthermore, we demonstrate that vanadate exerts the same effect on the UIC2 binding curves of the WT and the unilateral Walker A mutant Pgp variants. Since vanadate-dependent trapping is an extremely low-probability event, formation of the stable V_i_-trapped complex (Fig. 3), as well as the significant left shift of the UIC2 binding curves in the presence of V_i_ (Fig. 2b,c), can only be explained if single Walker A mutants are catalytically competent. Admittedly, this result is opposed to prevailing views insisting on the requirement of two intact nucleotide binding domains for ATP hydrolysis by Pgp^28^. While our experimental system has several limitations (binding and hydrolysis of ATP is measured indirectly, by monitoring the binding of a conformation sensitive antibody to a complex extracellular epitope of Pgp in semipermeabilized cells), we note that the majority of the studies reporting the inactivating effect of single Walker A mutations have been performed using heterologous expression systems such as Sf9^32^,^56^, Saccharomyces cerevisiae^57^ or purified and reconstituted proteins^52^,^57^,^58^. It is known that the plasma membrane composition influences the catalytic activity of ABC transporters, and that membrane cholesterol amounts influence Pgp activity^59^,^60^. Since membrane cholesterol levels are significantly lower in lower eukaryotes^60^,^61^, it may be that the low ATPase activity of the single Walker mutants was missed due to the different plasma membrane composition of the heterologous expression systems, or artefacts related to the solubilization, purification and reconstitution of the proteins^62^. We show that the single Walker A mutants show drug-stimulated nucleotide hydrolysis (Fig. 3 and Supplementary Fig. S2), decrease the intracellular accumulation of calcein (Fig. 5a), promote R123 efflux (Fig. 5d–e) and offer moderate protection against cytotoxic Pgp substrates (Supplementary Fig. S4). Taken together, our data indicate that, in contrast to prevailing views, single-site Walker A mutant Pgp molecules retain a weak, but significant uphill transport activity, a phenomenon which cannot be explained other than through coupling to repeated cycles of ATP hydrolysis.

Formation of the vanadate-trapped species occurs with comparable rates in WT and single mutant Pgp (Fig. 3), suggesting that the fraction of time the working transporter spends in the V_i_-sensitive posthydrolytic state is similar for WT and single mutant Pgp. Interestingly however, the rate of disassembly of the V_i_-trapped complex is greatly reduced in the single mutants (Fig. 4a). Assuming that the trapped complex harbors an ATP in the non-committed site, one might speculate that disassembly of the complex happens only upon eventual hydrolysis of that ATP. This process might happen at a rate of ~1 per hour in a non-committed wild-type catalytic center (cf., Fig. 4b), but never at all in a center disabled by a Walker A mutation (Fig. 4a). Taken together our experiments carried out with single Walker A mutants suggest that while one hydrolysis event is sufficient to reset the high-substrate affinity inward facing conformation in the absence of V_i_, dissociation of the V_i_-trapped complex also requires the hydrolytic activity of the contralateral site, supporting the possibility that the V_i_-trapped complex may not mimic a true catalytic transition state in case of Pgp. However, much further work will be required to fully understand these details.

Importantly, the fact that single Walker A mutations allow residual transport is incompatible with a model in which two ATP molecules must be hydrolyzed in each cycle. Our results are also difficult to reconcile with models suggesting that the two NBDs hydrolyze ATP in a strictly alternating order^28^,^47^. If the NBDs were indeed recruited in a strictly alternating fashion, every second ATP would have to be processed by the mutant catalytic center, causing the cycle to stall. Instead, our results indicate that the wild-type catalytic site can hydrolyze ATP in repeated cycles without hydrolysis at the other NBD. The simplest interpretation of our data is that in WT Pgp one of the two functionally equivalent sites becomes committed to hydrolysis in each cycle on a random basis, whereas in the single mutants commitment of the only functional site initiates every cycle.

**Figure 9:**
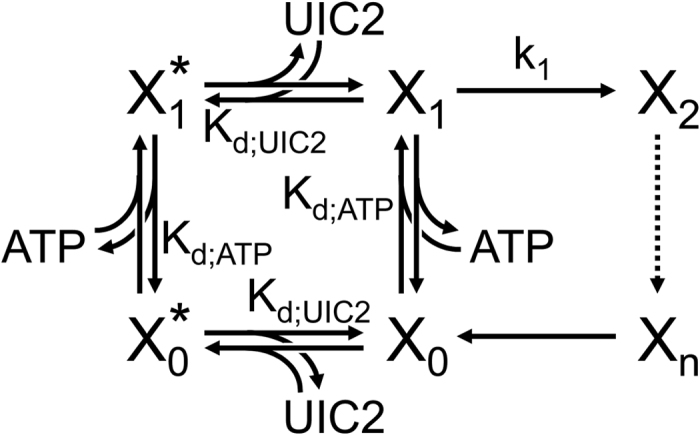
Simplified model of the catalytic cycle of Pgp that distinguishes inward- and outward-facing states.

Finally, a simplified kinetic model that distinguishes inward- and outward-facing states (Fig. 9; see full mathematical treatment in Supplementary Information) provides a semi-quantitative framework for interpretation of the data presented here, and offers some additional mechanistic insight. The model includes ATP-free (*X*_0_) and ATP-bound (*X*_1_) inward-facing states (associated with an open NBD dimer), and, for simplicity, depicts ATP binding as a single step assumed to be at rapid equilibrium. From the ATP-bound inward-facing conformation the transporter flips (with rate *k*_1_) to the ATP-bound outward-facing *X*_2_ state (associated with a closed NBD dimer). The model is very general, in that it does not specify (*dotted arrow*) the number and nature of intermediate states between *X*_2_ and the final post-hydrolytic outward-facing state *X*_n_, which eventually flips back to inward-facing state *X*_0_ upon NBD-dimer dissociation and release of hydrolysis products. Because UIC2 reactivity is believed to be uniformly low in all outward-facing states, the model allows UIC2 binding only to inward-facing conformations *X*_0_ and *X*_1_, yielding UIC2-bound states *X*_0_* and *X*_1_*, respectively. For simplicity, binding of UIC2 and ATP are assumed independent – consistent with the fact that UIC2 recognizes an extracellular epitope^22^ formed by the TMDs, the conformation of which is unlikely to sense mere binding of ATP to intracellular NBDs. The parameters of the model are as follows:

*K*_d;ATP_ is the dissociation constant of ATP from inward-facing Pgp,

*K*_d;UIC2_ is the dissociation constant of the UIC2 antibody from inward-facing Pgp,

*T*_1_ is the average life time of the inward-facing conformation,

i.e., of compound state {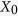; 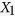; 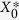; 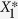},

*T*_1;min_ (=1/*k*_1_) is *T*_1_ in the presence of zero [UIC2] and saturating [ATP], and

*T*_2_ is the average life time of the outward-facing conformation, i.e., of compound state {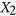;… 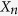}.

*v,* is the turnover rate for ATP hydrolysis (or substrate transport), which is the inverse of the total cycle time *T* (=*T*_1_ + *T*_2_).

Despite its simplicity, the model is congruent with a broad range of existing data. (i) Consistent with the literature^48^,^63^, the scheme (Fig. 9) predicts that ATP-hydrolysis and ATP-dependent substrate transport by Pgp follow Michaelis-Menten type kinetics (see Supplementary Information Eqs 3). (ii) It further predicts that UIC2 should act as a mixed type inhibitor of Pgp-mediated ATP-hydrolysis and substrate transport, by lowering the apparent *V*_*max*_ and by increasing the apparent *K*_*M*_ for ATP (see Supplementary Information Eqs 1, 2 and 5–6 for details). Indeed, UIC2 inhibits the efflux of Pgp substrates from multidrug resistant cells and significantly increases the cytotoxicity of Pgp-transported drugs^64^,^65^. (iii) Consistent with our finding that ATP shifts Pgp into a low UIC2-affinity conformation in a concentration dependent manner (Fig. 1a), the scheme (Fig. 9) predicts that increasing [ATP] should diminish UIC2-labeling (by a fixed concentration of UIC2) following a Michaelis-Menten-type dose-response relationship, and the apparent affinity of ATP for competing UIC2 labeling (*K*_A_) should be identical to the *K*_M_ of ATP for ATP-hydrolysis and substrate transport observable at the same fixed [UIC2] (see Supplementary Information Eq. 7). (iv) Finally, the model predicts that the apparent affinity for UIC2 binding (*K*_U_) should be sensitive to ATP concentration (see Supplementary Information Eqs 9–11), which was verified in UIC2 titration experiments (Supplementary Fig. S5). These features qualified the model to be suitable for semi-quantitative interpretation of our results, and allowed us to draw some additional mechanistic conclusions.

First, our experiments provide two independent estimates of the fraction of time wild-type Pgp spends in the outward- vs. inward facing conformations under normal, hydrolytic conditions in saturating ATP (i.e., the ratio *T*_2_/*T*_1;min_). The first approach builds on the maximal shift in apparent affinity for UIC2 binding (*K*_U_) caused by very high concentrations of ATP. In the experiments documented in Supplementary Fig. S5 we determined *K*_U_ at various different [ATP]. In the absence of ATP, *K*_U_ reflects the antibody’s dissociation constant (*K*_d;UIC2_), which was found to be ~2 μg/ml (~10 nM). In contrast, at very high [ATP], *K*_U_ approaches ~20 μg/ml ≈ 10*K*_d;UIC2_. This maximal ~10-fold shift in *K*_U_ by high [ATP] indicates that in saturating [ATP] Pgp spends 90% of the total cycle time in the outward-facing (UIC2-dim) conformation (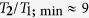, see Supplementary Information Eq. 11). An independent estimate of *T*_2_/*T*_1;min_ is obtained from the fraction of UIC2 label which resists even at saturating [ATP]. Applying a fixed [UIC2] of 10 μg/ml, equivalent to ≈5*K*_d;UIC2_, we found that approximately one third of the UIC2 label persists at very high [ATP] (Fig. 1a), from which Eq. 8 (see Supplementary Information) predicts a ratio *T*_2_/*T*_1;min_ of ~12. Thus, both approaches suggest that, in saturating ATP, WT Pgp spends the majority (>90%) of the total cycle time in the outward-facing conformation (compound state {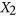;… 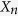} in the scheme (Fig. 9)). With other words, flipping from the ATP-bound inward-facing to the ATP-bound outward-facing conformation (step *X*_1_ → *X*_2_ in Fig. 9) is not the rate-limiting step of the overall cycle. A direct implication of this finding is that in Pgp substrate-mediated stimulation of ATPase turnover rate must reflect acceleration of some other step(s): mere speeding of step *X*_1_ → *X*_2_ could increase turnover rate by no more than ~10%. It will be interesting to establish how broadly this conclusion is applicable to other members of the ABC protein family. For instance, in the CFTR chloride ion channel (ABCC7) pore opening, believed to correspond to the transition from inward- to outward-facing, is no doubt the slowest step of the gating cycle, and is most robustly affected by channel phosphorylation, the primary mechanism for regulating CFTR activity (reviewed in^66^).

Second, the model allows comparison of the kinetics of the transport cycles of Walker A single mutants to that of WT. From the increased fraction of UIC2 label persisting in saturating ATP (~50%, Fig. 2b,c) we estimate a decreased ratio of 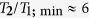 (Supplementary Information Eq. 8), suggesting that in saturating [ATP], the fraction of time spent in the outward-facing conformation is somewhat decreased for single Walker A mutants relative to WT. This explains on one hand the higher apparent affinities for substrate binding observed for the single mutants (Fig. 7b,c vs. Fig. 7a; bars labeled “control”), but in part also the more modest suppression of substrate affinities by 3–5 mM AMP-PNP (Fig. 8b; note also that for the single mutants 3–5 mM AMP-PNP is less than saturating (Fig. 2a and Supplementary Table S2)). In itself, the ~10 fold decrease in apparent affinity for ATP of the single mutants (Fig. 2b,c and Supplementary Table S2) would explain only a modest decrease in transport rate (to 80–90% of WT) at physiological ATP concentrations. Thus, to account for a transport rate which is only ~10–15% of WT (Fig. 5), the average overall cycle time *T* of the single mutants must be significantly prolonged even in saturating ATP. Given our above estimate of *T*_1;min_/*T*_2_ ≈ 10 for WT-Pgp, a mere increase in *T*_1;min_ (to yield 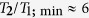), would prolong the overall cycle time *T* only by ~6%: this falls far short of explaining the robust decline in transport rate (see Supplementary Information for more details). Two possible scenarios could account for this discrepancy. One possibility is that both *T*_1;min_ and *T*_2_ are prolonged: in this view all cycles would go through ATP hydrolysis at the competent site, but both the rate of formation of the NBD dimer (*k*_1_) and the rate of ATP hydrolysis (step *X*_2_ → … *X*_n_, overall rate *k*_2_) would be slowed. Alternatively, besides allosterically slowing ATP hydrolysis at the competent site, the major disturbance caused by single mutations could be destabilization of the NBD dimer, i.e., an increase in the slow rate (*k*_-1_) of reverse step *X*_2_ → *X*_1_. As a consequence, only ~1 out of 6 sojourns in the *X*_2_ state would be terminated by ATP-hydrolysis (“productive” transport cycles), whereas in ~5 out of 6 cases the reverse pathway would be taken (“fruitless” partial cycles)–with little change in the cycle time *T* itself. A similar mechanism, i.e., ~20% coupling between ATP-hydrolysis and pore opening events, has been suggested for NBD1 Walker-A mutant K464A CFTR channels^67^.

In conclusion, we have shown that the high-to-low substrate affinity switch in Pgp coincides with the flipping of the TMDs to the outward-facing conformation, and that these events precede ATP hydrolysis. Double Walker A mutant Pgp is trapped in the high-affinity inward-facing conformation, but single mutants are capable of full transport cycles albeit with reduced efficiency. Release from the vanadate-trapped low-affinity outward-facing conformation requires two functional composite sites. These results support random recruitment of the two catalytic centers for ATP hydrolysis.

## Materials and Methods

### Chemicals

Cell culture media, supplements and chemicals were from Sigma-Aldrich (Budapest, Hungary). Fluorescent dyes including calcein acetoxymethyl ester (calcein-AM), BODIPY FL vinblastine (vinblastine-bodipy; VBL-BPY) and Alexa 647 succinimidyl ester were purchased from Life Technologies, Inc. (Carlsbad, CA, USA). The UIC2 and 15D3 anti-Pgp mAbs were prepared from hybridoma supernatants using affinity chromatography and were >97% pure by SDS/PAGE. Hybridoma cell lines were obtained from the American Type Culture Collections (Manassas, VA, USA). The UIC2 and 15D3 antibodies were labeled with Alexa 647 succinimidyl ester (A647) and separated from the unconjugated dye by gel filtration on a Sephadex G-50 column. The dye-to-protein labeling ratio was around 3 for each antibody preparation.

### Cell lines

The NIH 3T3 mouse fibroblast cell line was a kind gift from Michael Gottesman (National Institutes of Health, Bethesda, MD). The MDCK II Madin-Darby canine kidney cell line was obtained from Balazs Sarkadi (Molecular Biophysics Research Group, Hungarian Academy of Sciences, Budapest, Hungary). The cells were grown as monolayer cultures at 37 °C in an incubator containing 5% CO_2_, and were maintained by regular passage in Dulbecco’s modified Eagle’s medium (DMEM) supplemented with 10% heat-inactivated fetal calf serum, 2 mM L-glutamine, and 0.1 mg/ml penicillin-streptomycin cocktail.

### Vector constructs

*Sleeping Beauty* transposon vectors containing the wild-type (WT), K433M, K1076M or the K433M/K1076M double mutant human MDR1 cDNA were constructed as follows. Site-directed mutagenesis was performed using the QuikChange II Site-Directed Mutagenesis Kit (Agilent Technologies, Santa Clara, CA, USA) on pAcUW-LMDR1 vector carrying the wild-type human MDR1 cDNA. Mutations were generated according to the manufacturer’s instructions. The following oligonucleotide primers were used: 5′-AAACAGTGGCTGTG GGATGAGCACAACAGTCCAGCTGA-3′ and 5′-CAGCAGTGGCTGTGGGATGAGC ACAGTGGTCCAGCTCC-3′ for the K433M and K1076M mutations, respectively. The *Sleeping Beauty* (SB) transposon vector containing the cDNAs of EGFP and the puromycin resistance gene (PURO) in separate transcription cassettes, each driven by CAG promoter, was kindly provided by Dr. Tamás István Orbán (Hungarian Academy of Sciences, Budapest, Hungary). Full-length wild-type MDR1 cDNA was amplified by PCR from pAcUW-LMDR1 vector with the following pair of primers: 5′-TAGAATACCGGTAGGTCGGAATGGATCTTGAA-3′ and 5′-AGTGATGGATCCAACATCTCATACAGTCAGAG-3′ containing AgeI and BamHI restriction sites, respectively. The digested PCR product was cloned into the SB-EGFP-PURO transposon vector between the AgeI and BclI restriction sites, replacing EGFP, resulting in SB-MDR1-PURO vector. The PstI-BstBI restriction fragment of the MDR1 cDNA was then replaced in SB-MDR1-PURO with the mutated PstI-BstBI fragments derived from pAcUW-LMDR1K433M and pAcUW-LMDR1K1076M, resulting in SB-MDR1K433M-PURO and SB-MDR1K1076M-PURO vectors. The SB construct containing the K433M/K1076M double mutant MDR1 cDNA was generated by replacing the 3242bp long HindIII-HindIII fragment in SB-MDR1K1076M-PURO with that of SB-MDR1K433M-PURO, resulting in SB-MDR1K433M/K1076M-PURO. Full-length MDR1 cDNAs were sequenced and mutations were confirmed in all SB constructs.

### Establishment of transgenic cell lines

Cell lines stably expressing WT, K433M, K1076M, and K433M/K1076M double mutant MDR1/P-glycoprotein were established by the Sleeping Beauty (SB) transposon-based gene delivery system, using the 100 fold hyperactive SB transposase^68^. NIH 3T3 mouse fibroblast and MDCK II canine kidney cells were co-transfected with the SB transposase and SB transposon vector constructs by Lipofectamine2000 reagent (Life Technologies, Carlsbad, CA, USA), in accordance with the manufacturer’s instructions. Briefly, 3 × 10^5^ cells were seeded in 6-well-plates, 24 hours later cells were transfected with 2 μg vector DNA per well in a 10:1 ratio for the SB transposon and transposase constructs. 48 hours after transfection transgene positive cells were sorted by flow cytometry (FACS Aria High Speed Cell Sorter, Becton Dickinson) based on the cell surface expression of wild-type and mutant MDR1/P-glycoprotein. Protein expression was measured by antibody labeling using the human MDR1/P-glycoprotein specific monoclonal antibodies MRK16 (Abnova GmbH, Heidelberg, Germany) or 15D3. To obtain homogenously expressing cell populations, sorting procedure was repeated 2 weeks after transfection.

### Western blot analysis

Total cellular protein (2.5 μg/slot) was subjected to SDS-polyacrylamide gel electrophoresis on 8% polyacrylamide gel and electro-blotted to 0.45 μm pore size nitrocellulose membrane (GE Healthcare Life Sciences, Little Chalfont, Buckinghamshire, UK). Pgp expression was detected by a monoclonal anti-Pgp mAb (G-1, Santa Cruz Biotechnology Inc., Santa Cruz, CA, USA) and a goat anti-mouse HRP-conjugated IgG secondary antibody (Santa Cruz Biotechnology Inc., Santa Cruz, CA, USA), both applied at 1:5,000 dilution.

### Permeabilization of cells with Staphylococcus aureus α-toxin or streptolysin-O

*Staphylococcus aureus* α-toxin and streptolysin-O (Sigma-Aldrich, Budapest, Hungary) binds to the plasma membrane of cells and forms ring-structured toxin hexamers that are permeable for small water soluble molecules including nucleotides^69^. Cell suspensions (1 × 10^7^ cells/ml) were treated with 4 μg/ml α-toxin in phosphate-buffered saline (PBS) in the presence of 1% bovine serum albumin (BSA) at 37 °C for 30 min, allowing permeabilization of approximately 50% of the cells (judged by propidium iodide (PI) positivity)^25^. The reaction was stopped with 40 ml of 37 °C PBS and the cells were centrifuged for 7 min at 635 g at room temperature. Unbound toxin was removed by washing the cells 3 times with PBS and the cell pellet was re-suspended in PBS.

Cells grown attached to the bottom of Ibidi μ-Slide IV 0.4 (for confocal microscopy) were permeabilized by 50 μg/ml *Staphylococcus aureus* α-toxin or 20 μg/ml (approx. 1300 U/ml) streptolysin-O in the presence of 10 mM DTT and Protease Inhibitor Cocktail at 37 °C for 60 min in HEPES buffer (20 mM HEPES, 123 mM NaCl, 5 mM KCl, 1.5 mM MgCl_2_, 1 mM CaCl_2_) containing 1% FCS (allowing permabilization of 60–80% of cells).

### Determination of the apparent affinity of nucleotide binding

Permeabilized cells (1 × 10^6^ ml^−1^) were pre-incubated for 20 min with nucleotides added in a broad concentration range and then further incubated for 30 min with 10 μg/ml A647-conjugated UIC2 monoclonal antibody (all at 37 °C). UIC2 mAb binding to Pgp is a reversible reaction in the presence of ATP/Mg^2+^ (Supplementary Fig. S6), thus, UIC2 was applied at a quasi-saturating concentration (5*K*_d;UIC2_; Supplementary Fig. S5) and the duration of the incubation period was sufficient to reach equilibrium binding of UIC2 (Supplementary Fig. S6).

When ATP was applied under hydrolysis conditions the concentration of the cells was decreased to 1.5 × 10^4^ ml^−1^ in order to reduce the consumption of ATP. Under these conditions the activity of Pgp and endogenous ATPases present in the permeabilized cells did not significantly reduce the concentration of ATP in the samples (less than 3% of the ATP was converted to ADP).

To prevent ATP hydrolysis, ATP was added to permeabilized cells without Mg^2+^ in the presence of 5 mM EDTA. Alternatively, ATP was replaced by the non-hydrolysable ATP analogue AMP-PNP/Mg^2+^, or the experiment was conducted on ice. In the latter case the labeling of cells with A647-conjugated UIC2 monoclonal antibody was carried out at 4 °C for 45 min. Following antibody labeling, samples were washed 3 times with ice-cold PBS and centrifuged for 7 min at 635 g at 4 °C. The UIC2-A647 fluorescence intensity of the cells was measured by flow cytometry and plotted as a function of the nucleotide concentration. To determine the apparent affinity of Pgp to the nucleotides (*K*_*A*_) data points were fitted with the four-parameter Hill function, where *F*_*min*_ and *F*_*max*_ values are the minimum and maximum fluorescence intensities:


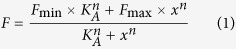


### Nucleotide trapping

The transition state of Pgp was stabilized by the addition of ATP/Mg^2+^ and vanadate (V_i_) or BeF_x_ at 37 °C. Permeabilized cells were incubated with 100 μM (for WT) or 500 μM (for K433M and K1076M) ATP/Mg^2+^ and 0.5 mM V_i_^70^ or BeF_x_ (200 μM BeSO_4_ and 1 mM NaF) in the presence or absence of substrates in PBS at 37 °C. 500 μl aliquots were taken at regular intervals and washed twice with 5 ml ice-cold PBS containing V_i_ or BeF_x_ at the same concentrations that were applied previously during incubation. Samples were re-suspended in ice-cold PBS and labeled with 10 μg/ml UIC2-A647 monoclonal antibody in the presence of the transition state analogue at 4 °C for 45 min. The UIC2-A647 fluorescence intensity of the samples (*F*) was plotted as a function of time (*t*). The *t*_*1/2*_ values representing the half-life of the UIC2-reactive Pgp conformation were calculated from an exponential fit of the data points according to the following equation:


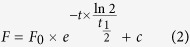


where *F*_*o*_ i.e. the difference between the zero and infinite time point of the curve and *c* is the background fluorescence intensity of the cells.

### Recovery from the V_i_-trapped post-hydrolysis state

Permeabilized cells trapped in the presence of ATP/Mg^2+^ (100 μM for wild-type and 500 μM for single Walker A mutants) and 0.5 mM V_i_ were washed 3 times with ice-cold PBS and incubated in the presence or absence of ATP and/or substrates (verapamil, vinblastine) at 37 °C. 500 μl aliquots containing 2 × 10^5^ cells were taken at regular intervals and washed twice with 5 ml PBS. The aliquots were incubated in the presence of 200 μM N-ethyl-maleimide ((NEM) to avoid de novo disulphide bond formation between Walker A cysteines^71^) and labeled with 10 μg/ml UIC2-A647 at 37 °C for 15 min or at 4 °C for 45 min. At the end of incubations the samples were washed three times with ice-cold PBS and kept on ice until measurement in the flow cytometer.

### Calcein assay

Calcein accumulation was measured as described^70^. Briefly, cells (0.25 × 10^6^ ml^−1^) were pre-incubated with a Pgp modulator cyclosporine A (CsA, 10 μM) for 10 min and then further incubated with 100 nM calcein-AM at 37 °C for 30 min. Finally, samples were washed with ice-cold PBS containing 1% FCS (FCS-PBS) and kept on ice until measurement. Dead cells stained with PI were excluded from the analysis. Transport activity was described by the ratio of median fluorescence intensities of the CsA-treated and untreated samples.

### Rhodamine 123 efflux assay

Cells (1 × 10^6^ ml^−1^) were pre-loaded with 0.5 μM rhodamine 123 (R123) for 30 min at 37 °C. Loading was terminated by chilling the tubes on ice; cells were washed three times with ice cold PBS (containing 8 mM glucose and 1% FCS, (gl-FCS-PBS), pH = 7.4). Efflux was initiated by re-suspending the cell pellet in gl-FCS-PBS pre-warmed to 37 °C. Efflux was monitored continuously over 5 minutes with a Becton Dickinson FACS Aria III flow cytometer at 37 °C using a temperature controlled sample injection chamber. Cells were further incubated at 37 °C and samples were taken at 10, 20, 30, 60 and 120 min time points and their median fluorescence intensity was measured using the same settings of the cytometer. Viable cells were selected by PI exclusion. Upon continuous monitoring of R123 efflux mean fluorescence intensities were computed for 10 second time intervals. Median fluorescence intensities were displayed as a function of time and the first order rate constants (*k*-values) were calculated from an exponential fit of the data points according to Eq. 3, where *a* is initial loading (i.e. the difference between the zero and infinite time point of the curve), *t* is the time in minutes, 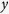 is mean fluorescence intensity at *t* time point, and *c* is background fluorescence intensity of the cells.


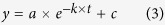


### Vinblastine-bodipy staining

Cells were plated in 8-well Ibidi μ-Slide (Ibidi GmbH, Martinsried, Germany) two days before the experiments to obtain nearly confluent cultures. The samples were pre-incubated with the following agents: Na-azide (10 mM) and 2-deoxy D-glucose (8 mM), V_i_ (2 mM) or NEM (50 μM) for 30 min and then further incubated with 100 nM vinblastine-bodipy (VBL-BPY) and 30 μg/ml A647-conjugated 15D3 anti-Pgp antibody for another 30 min at 37 °C.

### Flow cytometry

Calcein accumulation and R123 efflux measurements were carried out on a Becton Dickinson FACSAria^TM^ III Cell Sorter (Becton Dickinson, Mountain View, CA, USA). Calcein and R123 were excited by the 488 nm line of a solid state laser and the emitted light was detected using a 502 nm dichroic mirror and a 530/30 nm band-pass filter. PI was excited by the 562 nm line of a solid state laser and the emitted light was detected applying a 590 nm dichroic mirror and a 595/50 nm band-pass filter. In case of continuous monitoring of R123 efflux the sample injection chamber was kept at 37 °C, while in other cases it was at 4 °C. Cytofluorimetric data were analysed by using FCS Express 4 Research Edition (De Novo Software, Glendale, CA, USA).

UIC2-A647 labeling of permeabilized cells was measured by using a Becton Dickenson FACS Array flow cytometer; the data were analysed with the BDIS CellQuest software. A 635 nm laser was used for the excitation of the Alexa 647 dye and the fluorescence was detected in the red channel (661/16 nm), while the 532 nm laser was used for the excitation of PI (detected at 585/42 nm). Cell debris was excluded from analysis on the basis of FSC and SSC signals. The median fluorescence indicating UIC2 reactivity was determined in PI positive cells.

### Confocal laser scanning microscopy

Cellular localization of Pgp and VBL-BPY staining was studied by a three-laser confocal laser scanning microscope (Olympus FluoView 1000 confocal microscope based on an inverted IX-81 stand with an UPlansAPo 60 × NA 1.35 oil immersion objective, Hamburg, Germany). The 488-nm blue line of an argon-ion laser, and the 543-nm green and the 633-nm red helium-neon laser lines were used for the excitation of VBL-BPY, PI and Alexa 647, respectively. Fluorescence intensities were detected in the spectral ranges of 500–530 nm, 555–655 nm and 655–755 nm, respectively. To assess the co-localization of VBL-BPY (green) and Alexa 647-conjugated 15D3 (red) fluorescence signals, single optical slices of apical membrane surfaces were recorded for double-labeled cells. The pattern and extent of co-localization in the sections were determined by using the cross-correlation scattergrams of the green and red pixels of the images. Co-localization indices were determined from 30–50 cells (in three independent experiments). The Pearson’s co-localization index (CI) provides a reliable estimate on the extent of fluorescence co-localization: CI values close to zero indicate no or a very low degree, while CI ≥ 0.5 reflects a high degree of co-localization, whereas the CI = 1 value would correspond to a full overlap between the two colours in each pixel of the image.

In case of permeabilized cells VBL-BPY flurescence intensity was determined in pixels representing the apical plasma membrane selected on the basis of 15D3-Alexa-647 fluorescence intensity exceeding the threshold of 300 above background intensity.

### Statistical analysis

Data were analysed using SigmaStat (version 3.1, SPSS Inc., Chicago, IL, USA) and are presented as means ± SD. Comparison of two groups was carried out by unpaired *t*-test, statistical significance in the case of three or more groups was assessed using analysis of variance (ANOVA), applying the Holm-Sidak multiple comparison test for post hoc pair-wise comparison of the data. In the case of unequal variances Dunnett T3 post hoc pair-wise comparison method was used. Differences were considered significant at P < 0.05.

## Additional Information

**How to cite this article**: Bársony, O. *et al.* A single active catalytic site is sufficient to promote transport in P-glycoprotein. *Sci. Rep.* **6**, 24810; doi: 10.1038/srep24810 (2016).

## Supplementary Material

Supplementary Information
