# Supplementary material for: A single active catalytic site is sufficient to promote transport in P-glycoprotein
Source: Sci Rep. 2016 Apr 27;6:24810. doi: 10.1038/srep24810 (PMC4846820; doi:10.1038/srep24810)
Supplement: Supplementary Information [file srep24810-s1.pdf]

Title: A single active catalytic site is sufficient to promote transport in P-glycoprotein

Short title: A single intact NBD is sufficient for the activity of Pgp

Authors: Orsolya Bársony[1*], Gábor Szalóki[1*], Dóra Türk[2], Szabolcs Tarapcsák[1], Zsuzsanna Gutay-Tóth[1], Zsolt Bacsó[1], Imre J. Holb[3,4], Lóránt Székvölgyi[1], Gábor Szabó[1], László Csanády[5], Gergely Szakács[2,6†], Katalin Goda[1†]

[*] shared first authorship

[†] shared last authorship

Author affiliation: [1]Department of Biophysics and Cell Biology, University of Debrecen, P.O. Box 400, Debrecen H-4002, Hungary and [2]Institute of Enzymology, Research Centre for Natural Sciences, Hungarian Academy of Sciences, [3]Institute of Horticulture, University of Debrecen, P.O. Box 36, H-4015 Debrecen, Hungary, [4]Plant Protection Institute, Centre for Agricultural Research, Hungarian Academy of Sciences, H-1525 Budapest, Hungary, [5]MTA-SE Ion Channel Research Group, Budapest H-1094, Hungary and [6]Institute of Cancer Research, Department of Medicine I, Comprehensive Cancer Center, Medical University of Vienna, Vienna, Austria

Corresponding authors: Correspondence should be addressed to Katalin Goda, Department of Biophysics and Cell Biology, Faculty of Medicine, University of Debrecen, Debrecen, Hungary; Phone: 36-52-412-623; E-mail:goda@med.unideb.hu and Gergely Szakács, Institute of Enzymology, Research Centre for Natural Sciences, Hungarian Academy of Sciences, Budapest, Hungary; Phone: 36-1-382-6715; E-mail:szakacs.gergely@ttk.mta.hu

Supplementary Figures

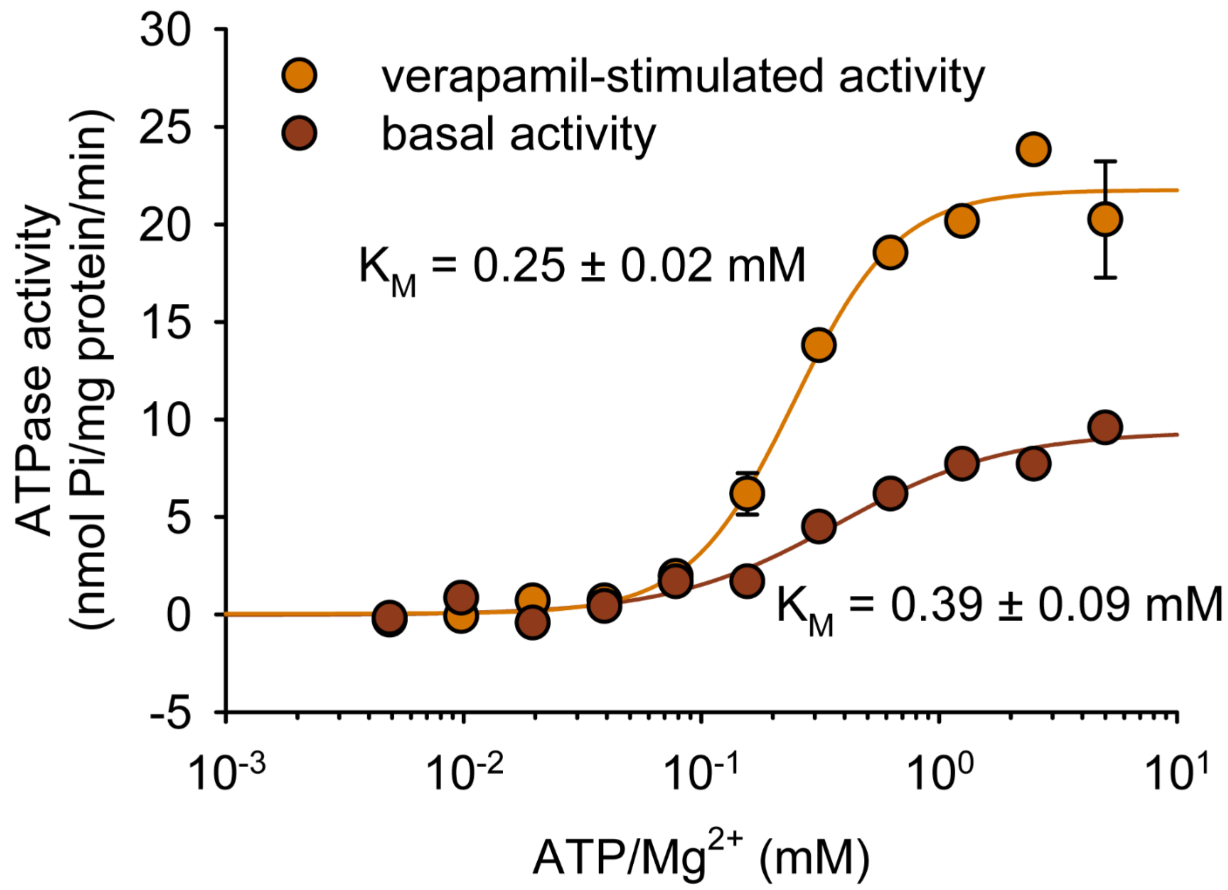

Supplementary Fig. S1: Basal and 10 μM verapamil-stimulated ATPase activity measured in a membrane sample prepared from NIH 3T3 cells expressing WT human Pgp at extremely high levels (Pgp density >10^6/cell). High level expression of Pgp was achieved by continuous selection of the transfected cells in 700 nM doxorubicin**.** The vanadate-sensitive ATPase activity (i.e. difference between the ATPase activities measured in the absence and the presence of 100 μM vanadate) was determined by measuring the inorganic phosphate liberation using a colorimetric assay[1]. The data points are means of three parallel samples (±SD), the K_M values for ATP hydrolysis are calculated from three independent measurements.

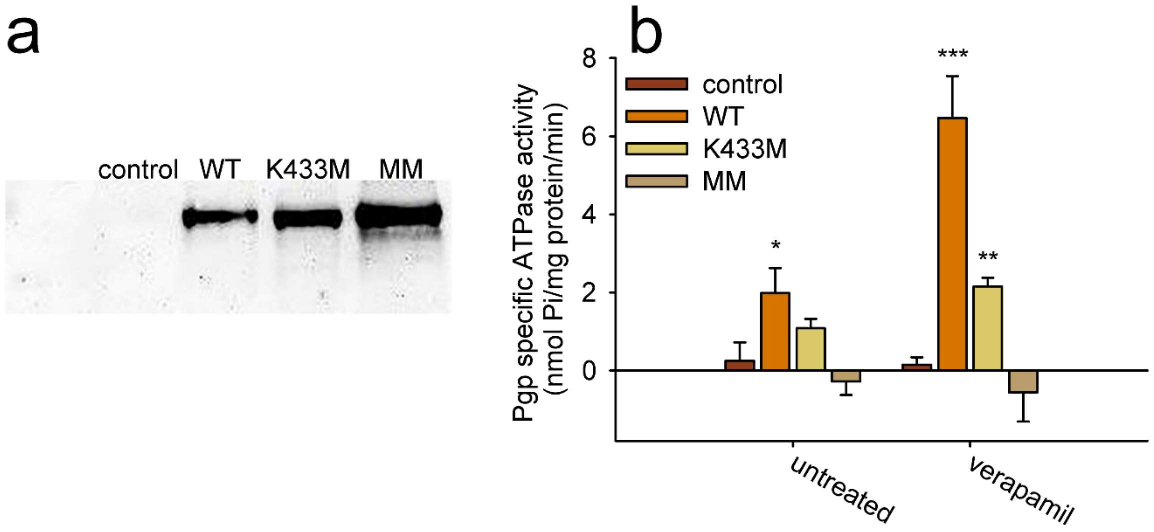

Supplementary Fig. S2: ATPase activity measurements

a. Western blot of membrane vesicles prepared from NIH 3T3 cells expressing wild-type (WT), K433M or K433M/K1076M (MM) Pgp. NIH 3T3 is shown as a control. The cell lines stably expressing WT and mutant Pgp variants were established by transfection with the Sleeping Beauty transposon-based gene delivery system and subsequent sorting by flow cytometry (FACS Aria High Speed Cell Sorter, Becton Dickinson) based on the cell surface expression of Pgp detected by Alexa 647 conjugated 15D3 anti-Pgp mAb (see Materials and Methods). To obtain homogenously expressing cell populations with high Pgp expression levels the sorting procedure was repeated 3 to 5 times (drug selection was not an option, because of the induced expression of the endogenous murine Abcb transporters). Compared to the doxorubicin selected cells shown in Fig S1, we could only achieve a 25-30 % expression level in the case of the K433M variant, while the double mutant variant (MM) showed ~ 100 % expression level. The immunoblot was developed by the human Pgp specific mouse monoclonal antibody G-1 (see Materials and Methods).

b. Pgp-specific ATPase activity. To increase the signal to noise ratio of the ATPase assay, values were corrected for the relatively high background activity associated with the endogenous ATPases. Thus, Pgp-specific ATPase activity is defined as the difference between the vanadate sensitive ATPase activities measured in the absence and the presence of the Pgp inhibitor cyclosporine A (10 μM). The ATPase activities were determined by measuring the inorganic phosphate liberation using a colorimetric assay[1]. Bars represent the means (±SD) of three independent measurements performed with six parallel samples. Significant differences compared to untreated NIH 3T3 control membrane are shown by ***: $P<0.001$, **: $P<0.01$, *: $P<0.05$.

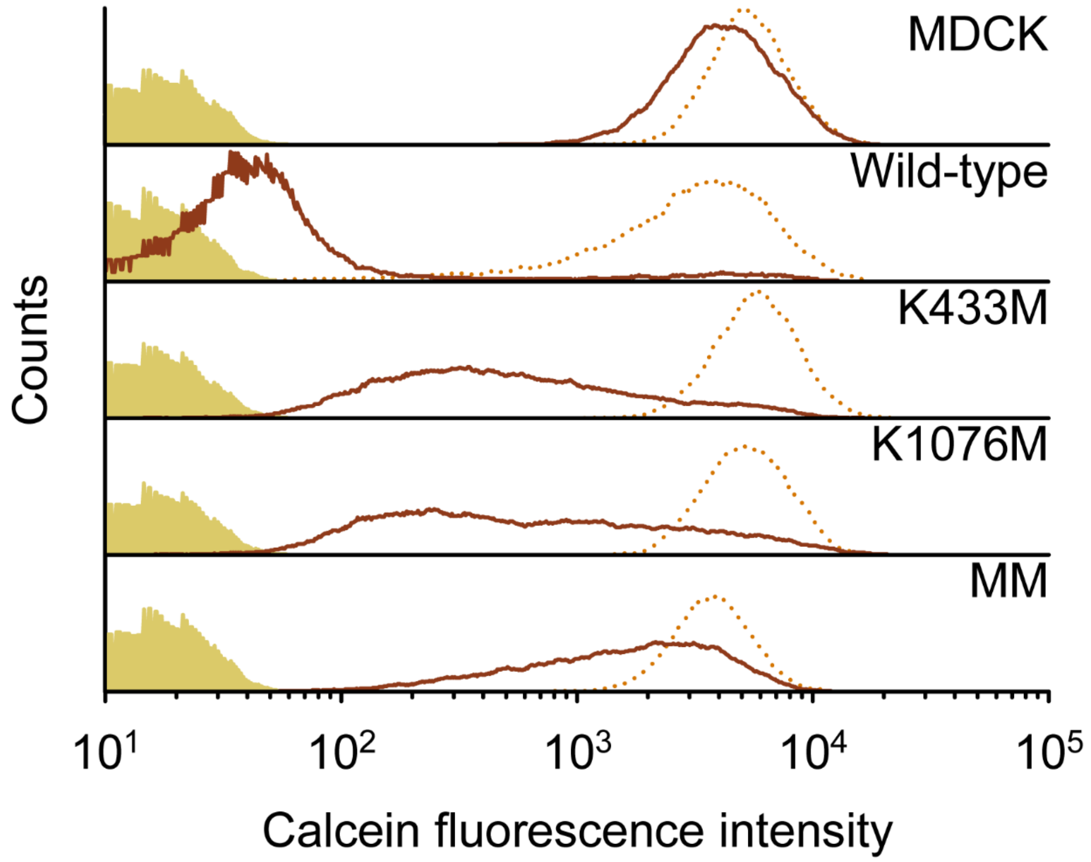

Supplementary Fig. S3: Representative fluorescence intensity distribution histograms from flow cytometric calcein accumulation measurements. Cells were incubated with 0.1 µM calcein-AM in the presence (*dotted line*) or absence (*brown line*) of the Pgp-inhibitor cyclosporin A (CsA, 10 µM) for 30 minutes. Background fluorescence intensity of the unstained cells is represented by *ocher histograms*.

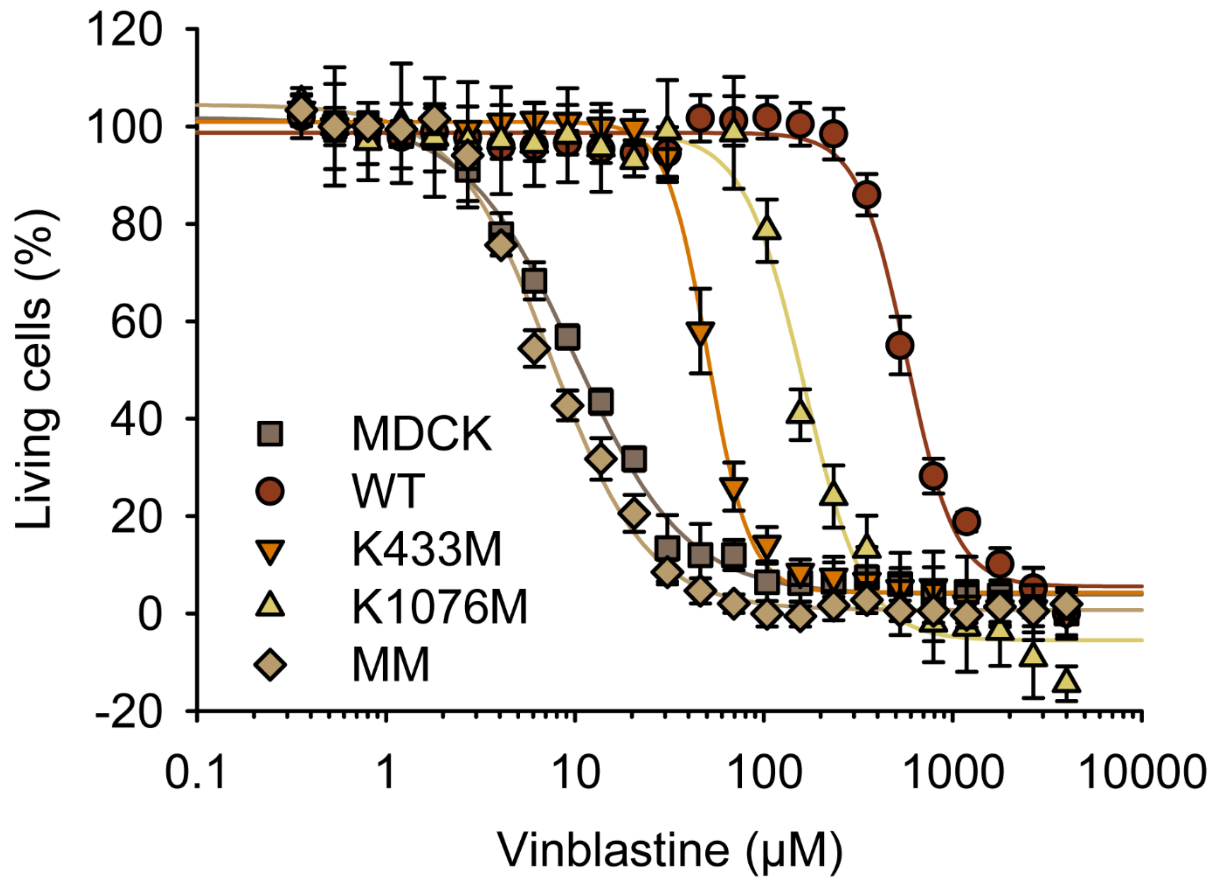

Supplementary Fig. S4: Cytotoxic effect of vinblastine on MDCK cells expressing wild-type, single or double Walker A mutant Pgp at comparable level and their non-transfected counterpart. Cells were seeded in 96-well plates at a cell density of 5×10^3 cells/well. 24 hours later vinblastine was added at different concentrations and the plates were further incubated for 72 h at 37 ºC. The cell viability was determined using the AlamarBlue assay (Serotec, UK) measuring the 530/590 nm fluorescence intensity of the dye in an automated microplate reader (BioTec Synergy HT, US). The fluorescence intensities of the samples were normalized to the fluorescence of the vinblastine untreated control sample, and plotted as a function of vinblastine concentration. The data points are means of eight parallel samples (±SD) from a representative experiment out of three.

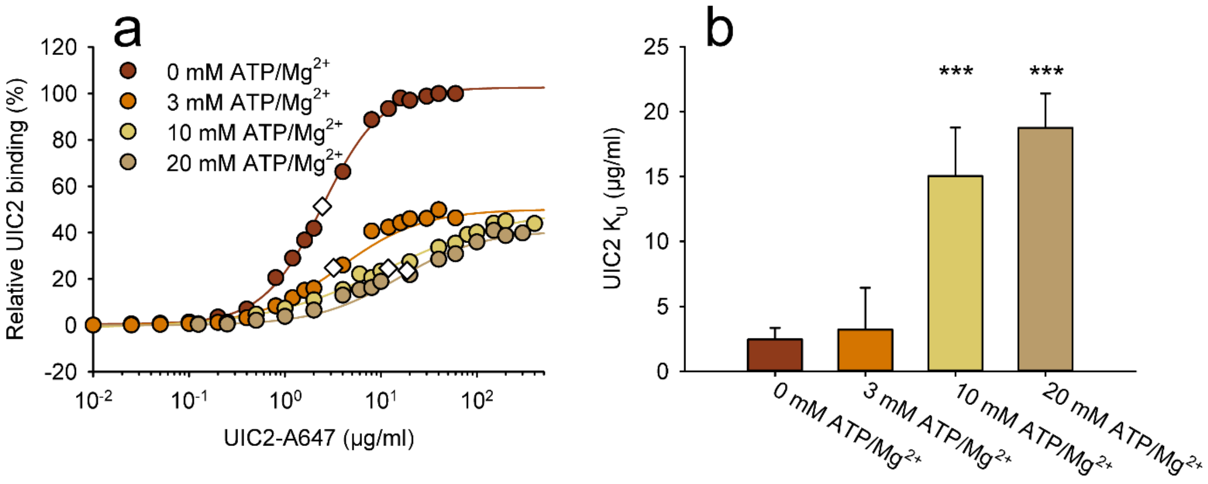

Supplementary Fig. S5: (a) Representative UIC2 mAb binding curves measured at different ATP concentrations in permeabilized wild-type Pgp expressing NIH 3T3 cells and (b) the K_U values of UIC2 mAb (apparent affinities of UIC2 mAb binding at different ATP concentrations). K_U values were calculated on the basis of at least three independent UIC2 mAb titration experiments (means ± SD; significant differences compared to the 0 mM ATP-treatment are shown by ***, (P<0.001)).

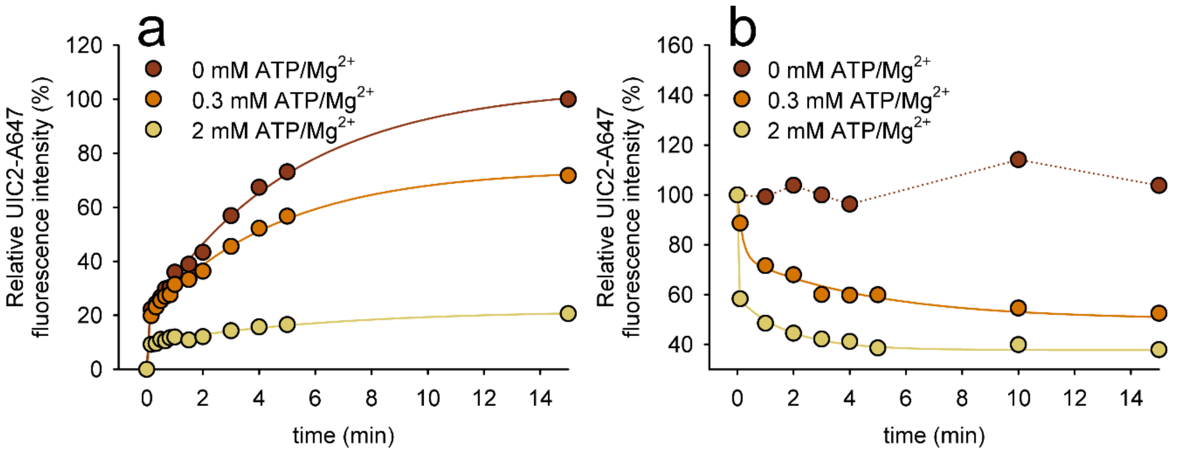

Supplementary Fig. S6. Binding (a) and dissociation (b) kinetics of UIC2 antibody measured in permeabilized, wild-type Pgp expressing NIH 3T3 cells at different ATP concentrations. The fluorescence intensities were normalized to the maximal binding of UIC2 mAb measured in the presence of no ATP. Data are shown for one representative experiment out of three independent experiments.

Supplementary Tables

Nucleotide	Condition (Figure 1.)	K_A (mM, ± SD)	Hill slope (± SD)	Assumed catalytic step(s) in the ATP hydrolysis cycle
ATP (n=59)	37 °C, Mg^{2+} (Fig. 1A)	0.25 ± 0.16	0.77 ± 0.20	hydrolysis
ATP (n=3)	37 °C, no Mg^{2+} (Fig. 1B)	1.92 ± 0.33	1.60 ± 0.22	nucleotide binding and NBD dimerization
ATP (n=7)	4 °C, Mg^{2+} (Fig. 1D)	2.70 ± 1.83	0.66 ± 0.17	nucleotide binding and NBD dimerization
AMP-PNP (n=17)	37 °C, Mg^{2+} (Fig. 1C)	0.27 ± 0.14	1.75 ± 0.34	nucleotide binding and NBD dimerization
ATP +V_i (n=11)	37 °C, Mg^{2+} (Fig. 1A)	0.07 ± 0.04	0.92 ± 0.15	post-hydrolysis state

Supplementary Table S1. Summary of K_A (apparent nucleotide affinity) and Hill slope values of various nucleotides for competing UIC2 labeling, obtained for wild-type human Pgp.

Nucleotide	Condition	K_A		Hill slope	
		K433M	K1076M	K433M	K1076M
ATP	37 °C, Mg^{2+}	2.08 ± 1.04<br>$P < 0.001$<br>(n=16)	2.03 ± 0.91<br>$P < 0.001$<br>(n=6)	0.90 ± 0.32<br>$P = 0.158$<br>(n=16)	0.89 ± 0.33<br>$P = 0.452$<br>(n=6)
AMP-PNP	37 °C, Mg^{2+}	3.40 ± 1.42<br>$P < 0.001$<br>(n=4)	3.01 ± 0.92<br>$P < 0.001$<br>(n=4)	2.06 ± 0.29<br>$P = 0.261$<br>(n=4)	1.68 ± 0.23<br>$P = 0.698$<br>(n=4)
ATP + V_i	37 °C, Mg^{2+}	0.44 ± 0.28<br>$P = 0.002$<br>(n=11)	0.80 ± 0.33<br>$P < 0.001$<br>(n=5)	1.08 ± 0.24<br>$P = 0.257$<br>(n=11)	1.03 ± 0.28<br>$P = 0.582$<br>(n=5)

Supplementary Table S2. Summary of K_A (apparent nucleotide affinity) and Hill slope values of various nucleotides for competing UIC2 labeling, obtained for single Walker A mutant Pgps (K433M and K1076M). The *P* values were obtained from one way ANOVA followed by Holm-Sidak post-hoc test for statistical comparisons of mean values to those of the wild-type cells shown in Supplementary Table S1.

Treatment	$t_{1/2}$ of BeF_x trapping		
	Wild-type Pgp	K433M	K1076M
**Control**	2.31 ± 0.69 (n=10)	1.92 ± 0.85 (n=8)	3.36 ± 2.83 (n=7)
**+ Verapamil (50 µM)**	0.38 ± 0.26 (n=5) $P < 0.001$	0.34 ± 0.11 (n=5) $P < 0.001$	0.29 ± 0.08 (n=6) $P < 0.012$

Supplementary Table S3. The effect of verapamil on the kinetics of the BeF_x-trapping reaction as reflected by the transition from the high- to low UIC2-affinity conformation. The $t_{1/2}$ values represent the half-life of the Pgp conformation which binds UIC2 with high affinity (see Materials and Methods). The *P* values were obtained from Student's *t* test for statistical comparisons of $t_{1/2}$ of the verapamil-treated and untreated control samples (one-tailed test). The $t_{1/2}$ values of the single mutants do not differ significantly from the wild-type either in case of the control (P=0.229) or the verapamil treated samples (P=0.669) by ANOVA.

Simplified kinetic model distinguishing inward- and outward-facing states of Pgp

Description of the model:

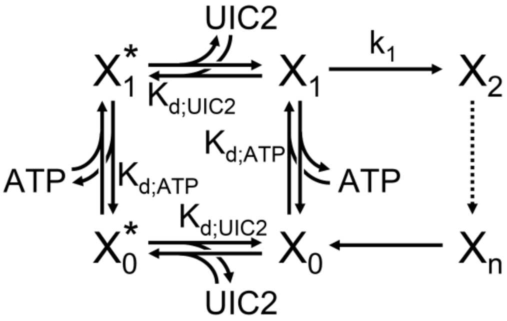

Scheme 1

This simplified model includes ATP-free (X_0) and ATP-bound (X_1) inward-facing states (presumably associated with an open NBD dimer), and, for simplicity, depicts ATP binding as a single step assumed to be at rapid equilibrium. From the ATP-bound inward-facing conformation the transporter flips (with rate k_1) to the ATP-bound outward-facing X_2 state (presumably associated with a closed NBD dimer). The model is very general, in that it does not specify (*dotted arrow*) the number and nature of intermediate states between X_2 and the final post-hydrolytic outward-facing state X_n, which eventually flips back to inward-facing state X_0 upon NBD-dimer dissociation and release of hydrolysis products. The model further assumes that UIC2 can bind only to inward-facing conformations X_0 and X_1, yielding UIC2-bound ATP-free and ATP-bound states X_0* and X_1*, respectively. For simplicity, binding of UIC2 and ATP are assumed independent – consistent with the fact that UIC2 recognizes an extracellular epitope[2], the conformation of which is unlikely to sense mere binding of ATP to intracellular NBDs. The parameters of the model are as follows:

$K_{d;ATP}$ is the dissociation constant of ATP from inward-facing Pgp,

$K_{d;UIC2}$ is the dissociation constant of the UIC2 antibody from inward-facing Pgp,

T_1 is the average life time of the inward-facing conformation, i.e., of compound state { X_0 ; X_1 ; X_0^*; X_1^*},

$T_{1;\min}$ (= $1/k_1$) is T_1 in the presence of zero [UIC2] and saturating [ATP], and

T_2 is the average life time of the outward-facing conformation, i.e., of compound state { X_2 ;… X_n }.

Note that the turnover rate (v) for ATP hydrolysis (or substrate transport) is the inverse of the total cycle time T (=T_1+T_2).

Validation of the model:

1. Consistent with the literature[3,4], Scheme 1 predicts that ATP-hydrolysis and ATP-dependent substrate transport by Pgp follow Michaelis-Menten type kinetics. The K_{M} and $V_{\max}$ values are given by

$$K_{\mathrm{M}} = \frac{K_{\mathrm{d;ATP}}}{1+\frac{K_{\mathrm{d;UIC2}}}{[\mathrm{UIC2}]+K_{\mathrm{d;UIC2}}}\cdot\frac{T_2}{T_{1;\min}}} \quad \text{and} \tag{1}$$

$$V_{\max} = \frac{1}{\left(1+\frac{[\mathrm{UIC2}]}{K_{\mathrm{d;UIC2}}}\right)\cdot T_{1;\min}+T_2} \ . \tag{2}$$

2. Also consistent with the literature[5-7], the model predicts that UIC2 should act as a mixed type inhibitor of Pgp-mediated ATP-hydrolysis and substrate transport, both by lowering the apparent V_{max} and by increasing the apparent K_M for ATP. In the absence of UIC2 these parameters are given by

$$K_{\mathrm{M}}\left([\mathrm{UIC2}]=0\right) = \frac{K_{\mathrm{d;ATP}}}{1+T_2/T_{1;\min}} \quad \text{and} \tag{3}$$

$$V_{\max}\left([\text{UIC2}]=0\right)=\frac{1}{T_{1;\min}+T_2}=\frac{1}{1/k_1+T_2}\ , \quad (4)$$

whereas at very high [UIC2] they asymptotically approach

$$\lim_{[\text{UIC2}]\to\infty} K_{\text{M}} = K_{\text{d;ATP}}\text{, and} \quad (5)$$

$$\lim_{[\text{UIC2}]\to\infty} V_{\max} = 0\,. \quad (6)$$

3. The model further predicts that ATP should compete with fractional UIC2-labeling (Y) of Pgp in a dose-dependent manner (as it is shown in Supplementary Fig. S5).

3.1. In particular, consistent with the data in Figure 1a, at a fixed concentration of UIC2 increasing [ATP] should diminish UIC2-labeling from Y_0 (fractional labeling at zero [ATP]) to Y_∞ (fractional labeling at saturating [ATP]) following a Michaelis-Menten-type dose-response relationship. The apparent affinity of ATP for competing UIC2 labeling (K_A) should be identical to the K_M of ATP for ATP-hydrolysis and substrate transport observable at the same fixed [UIC2], i.e.,

$$K_{\text{A}}=\frac{K_{\text{d;ATP}}}{1+\dfrac{K_{\text{d;UIC2}}}{[\text{UIC2}]+K_{\text{d;UIC2}}}\cdot\dfrac{T_2}{T_{1;\min}}}\left(=K_{\text{M}}\right), \quad (7)$$

whereas residual UIC2 labeling at saturating [ATP] obeys the equation

$$\frac{Y_\infty}{Y_0}=\frac{1}{1+\dfrac{K_{\text{d;UIC2}}}{[\text{UIC2}]+K_{\text{d;UIC2}}}\cdot\dfrac{T_2}{T_{1;\min}}}\,. \quad (8)$$

3.2. Similarly, consistent with the data in Supplementary Fig. S5, the apparent affinity for [UIC2] binding (K_U) should be sensitive to ATP concentration. At a fixed [ATP] the apparent affinity for UIC2 binding is given by

$$K_{\mathrm{U}} = K_{\mathrm{d;UIC2}} \cdot \left(1 + \frac{[\mathrm{ATP}]}{[\mathrm{ATP}] + K_{\mathrm{d;ATP}}} \cdot \frac{T_2}{T_{1;\mathrm{min}}} \right). \tag{9}$$

Thus, at zero [ATP]

$$K_{\mathrm{U}} \left([\mathrm{ATP}] = 0 \right) = K_{\mathrm{d;UIC2}}, \tag{10}$$

whereas at very high [ATP] K_U asymptotically approaches

$$\lim_{[\mathrm{ATP}] \to \infty} K_{\mathrm{U}} = K_{\mathrm{d;UIC2}} \cdot \left(1 + T_2 / T_{1;\mathrm{min}} \right). \tag{11}$$

4. Under non-hydrolytic conditions, e.g., using AMP-PNP or 0 Mg^{2+}, Pgp can perform only partial cycles. Because the pathway from state X_2 to state X_n (dotted arrow in model) is blocked, the transporter returns to its inward-facing conformation presumably through slow reversal of step $X_1 \rightarrow X_2$. (Because of its slow rate, this reverse transition is neglected in the scheme describing normal, hydrolytic cycles.) Importantly, all the statements in sections 2. and 3. above, including all the equations, identically apply under such conditions, with the understanding that T_2 now reflects the life time of the outward-facing conformation in the absence of hydrolysis. This allows interpretation of the data in Figs. 1b-d and 2a.

Quantitative predictions of the model:

1. Quantitative predictions on the kinetics of the WT-Pgp transport cycle

1.1. Two observable parameters provide two independent ways to estimate the fraction of time the transporter spends in the outward- vs. inward-facing conformations under normal, hydrolytic conditions in saturating ATP (i.e., the ratio $T_2/T_{1;\min}$).

1.1.1. Using the apparent affinities for [UIC2] binding (Supplementary Fig.S5)

At zero [ATP] $K_{\mathrm{U}}\left([\mathrm{ATP}]=0\right)=K_{\mathrm{d;UIC2}}\approx 2$ μg/ml (~ 10 nM) . In contrast, at very high [ATP] K_{U} approaches ~20 mg/ml ≈ $10K_{\mathrm{d;UIC2}}$. Thus, using Eq. 11, $10\approx 1+T_2/T_{1;\min}$, from which $T_2/T_{1;\min}\approx 9$.

1.1.2. Using the fraction of UIC2 label which resists even very high [ATP] (Fig. 1a)

Applying a fixed [UIC2] of 10 mg/ml, equivalent to ≈ $5K_{\mathrm{d;UIC2}}$, we found that approximately one third of the UIC2 label resists even very high [ATP]. Thus, substituting into Eq. 8, $\frac{1}{3}\approx\frac{1}{1+\frac{1}{5+1}\cdot\frac{T_2}{T_{1;\min}}}$, from which $T_2/T_{1;\min}\approx 12$.

Both approaches predict that, in saturating ATP, WT-Pgp spends the majority (>90%) of the total cycle time in the outward-facing conformation (compound state $\{X_2;\ldots\ X_n\}$ in Scheme 1). With other words, flipping from the ATP-bound inward-facing to the ATP-bound outward-facing conformation (step $X_1\rightarrow X_2$ in Scheme 1) is not the rate-limiting step of the overall cycle.

1.2. ATP-binding affinity to inward-facing Pgp

Eq. 7 allows estimation of $K_{\mathrm{d;ATP}}$ using the estimated ratio $T_2/T_{1;\min}$ and the measured apparent affinity for competing UIC2 labeling (K_{A}). Thus, at [UIC2] ≈ $5K_{\mathrm{d;UIC2}}$ we measured

K_A=0.25 mM (Fig. 1a and Supplementary Table S1). Substituting this, and $T_2 / T_{1;\min} \approx 10$ (from 1.1., above), into Eq. 7, $0.25 \text{ mM} = \frac{K_{\text{d;ATP}}}{1+\frac{1}{5+1}\cdot 10}$, from which $K_{d;ATP} \approx 0.7$ mM. Further, from Eq.3, the predicted K_M for ATP-hydrolysis and substrate transport in the absence of UIC2 is $K_M\left([\text{UIC2}]=0\right)=\frac{0.7 \text{ mM}}{1+10}\approx 60\ \mu\text{M}$

1.*3. Behaviour in AMP-PNP*

Similar apparent affinities and nucleotide-resistant UIC2-fractions when using ATP or AMP-PNP (Fig. 1a, c and Supplementary Table S1) suggest that the binding affinity for AMP-PNP to inward-facing Pgp is similar to that of ATP, and that the ratio $T_2/T_{1;\min}$ is also similar for the two nucleotides, despite the fact that AMP-PNP-driven conformational changes are restricted to partial cycles. Because in ATP the rate of the $X_2 \rightarrow X_1$ step is likely much slower than rate $X_2 \rightarrow ... X_n$, in AMP-PNP T_2 is likely prolonged. Thus, a plausible explanation is that in AMP-PNP both T_2 and $T_{1;\min}$ are comparably prolonged.

2. Quantitative predictions on the kinetics of the single-mutant KM- and MK-Pgp transport cycle

2.1. Using the fraction of UIC2 label which resists even very high [ATP] (Fig. 2b-c) allows estimation of the fraction of time single-mutant transporters spends in the outward- vs. inward-facing conformations under normal, hydrolytic conditions in saturating ATP (i.e., ratio $T_2/T_{1;\min}$).

Applying a fixed [UIC2] of 10 mg/ml, equivalent to ≈ $5K_{d;UIC2}$, we found that approximately one half of the UIC2 label resists even very high [ATP]. Thus, substituting into Eq. 8, $\frac{1}{2} \approx \frac{1}{1+\frac{1}{5+1}\cdot\frac{T_2}{T_{1;min}}}$, from which $T_2/T_{1;min} \approx 6$.

This calculation suggests that, in saturating ATP, the fraction of time spent in the outward-facing conformation (compound state $\{X_2;\ldots X_n\}$ in Scheme 1) is decreased for KM and MK single-mutants relative to WT: i.e., either T_2 is decreased, or $T_{1;min}$ is increased. Considering the fact that the transport rate of the single mutants is only ~10-15% of WT (Fig. 5), average overall cycle time T should be prolonged. Thus, it would be plausible to conclude that $T_{1;min}$ is increased in the single mutants (i.e., rate k_1 decreased). However, given our above estimates of $T_{1;min} \approx 10$ ms and $T_2 \approx 100$ ms for WT-Pgp, a mere increase in $T_{1;min}$ from ~10 ms to ~17 ms (to yield $T_2/T_{1;min} \approx 6$), would prolong overall cycle time T only by ~6% (from 110 ms to 117 ms): this falls far short of accounting for the robust decline in transport rate. Two possible scenarios could account for this discrepancy. One possibility is that both $T_{1;min}$ and T_2 are prolonged: a choice of $T_{1;min} \approx 100$ ms, $T_2 \approx 600$ ms would yield a turnover rate ~6-fold lower than WT, with $T_{1;min}/T_2 \approx 6$, as observed. Alternatively, the major disturbance in the single mutants could be an increase in the rate (k_{-1}) of reverse step $X_2 \rightarrow X_1$ (i.e., destabilization of the pre-hydrolytic NBD dimer), such that only ~1 out of 6 sojourns in the X_2 state ("fruitful" transport cycles) were terminated by ATP-hydrolysis (pathway $X_2 \rightarrow \ldots X_n \rightarrow X_0$; overall rate k_2), whereas in ~5 out of 6 cases the reverse pathway were taken ("unfruitful" partial cycles) – with little change in the cycle time T itself. (Suitable rates for this view could be, e.g., k_1=100 s^{-1}, k_{-1}=0.8 s^{-1}, k_2=9.2 s^{-1} for WT, yielding $T_{1;min}$ = 10 ms and T_2 = 100 ms with 92% coupling, but k_1=59 s^{-1}, k_{-1}=8.3 s^{-1}, k_2=1.7 s^{-1} for the single mutants, yielding $T_{1;min}$ = 17 ms and T_2 = 100 ms with 17% coupling.) Note, that a similar mechanism,

i.e., ~20% coupling between ATP-hydrolysis and pore opening events, has been suggested for NBD1 Walker-A mutant K464A CFTR channels [8].

2.2. ATP-binding affinity to inward-facing single-mutant Pgps

Eq. 7 allows estimation of $K_{d;ATP}$ using the ratio $T_2/T_{1;min}$ and the measured apparent affinity for competing UIC2 labeling (K_A). Thus, at [UIC2] ≈ 5$K_{d;UIC2}$ we measured K_A≈2.5 mM (Fig. 2b, c and Supplementary Table S2). Substituting this, and $T_2 / T_{1;min} \approx 6$ (from 1.1., above), into Eq. 7, $2.5 \text{ mM} = \frac{K_{d;ATP}}{1+\frac{1}{5+1}\cdot 6}$, from which $K_{d;ATP} \approx 5$ mM. This, about an order of magnitude, decrease in ATP binding affinity in the Walker-A single-mutants. Further, from Eq.3, the predicted K_M of the single-mutants for ATP-hydrolysis and substrate transport in the absence of UIC2 is $K_M\left([\text{UIC2}]=0\right)=\frac{5 \text{ mM}}{1+6}\approx 0.7 \text{ mM}$.

2.3. Behaviour in AMP-PNP

Similar apparent affinities and nucleotide-resistant UIC2-fractions when using ATP or AMP-PNP (Fig. 2a vs. b-c and Supplementary Table S1) suggest that the binding affinity for AMP-PNP to inward-facing single-mutant Pgp is again similar to that of ATP, and that the ratio $T_2/T_{1;min}$ is also similar for the two nucleotides.

APPENDIX

1. Turnover rate for ATP-hydrolysis and substrate transport

$$v = \frac{1}{T} = \frac{1}{T_1 + T_2}$$

Assuming rapid equilibrium for ATP binding

$$\begin{aligned}\frac{1}{T_1} &= k_1 \cdot \frac{\left[X_1\right]}{\left[X_0\right]+\left[X_1\right]+\left[X_0^*\right]+\left[X_1^*\right]} = \\ &= k_1 \cdot \frac{\left[X_1\right]+\left[X_1^*\right]}{\left[X_0\right]+\left[X_1\right]+\left[X_0^*\right]+\left[X_1^*\right]} \cdot \frac{\left[X_1\right]}{\left[X_1\right]+\left[X_1^*\right]} = \\ &= k_1 \cdot \frac{[\mathrm{ATP}]}{[\mathrm{ATP}]+K_{\mathrm{d;ATP}}} \cdot \frac{K_{\mathrm{d;UIC2}}}{[\mathrm{UIC2}]+K_{\mathrm{d;UIC2}}}\end{aligned}$$

from which

$$\begin{aligned}v &= \frac{1}{\frac{[\mathrm{UIC2}]+K_{\mathrm{d;UIC2}}}{k_1 K_{\mathrm{d;UIC2}}} \cdot \frac{[\mathrm{ATP}]+K_{\mathrm{d;ATP}}}{[\mathrm{ATP}]} + T_2} = \\ &= \frac{k_1 K_{\mathrm{d;UIC2}}[\mathrm{ATP}]}{\left([\mathrm{UIC2}]+K_{\mathrm{d;UIC2}}\right)\left([\mathrm{ATP}]+K_{\mathrm{d;ATP}}\right)+k_1 K_{\mathrm{d;UIC2}} T_2 [\mathrm{ATP}]} = \\ &= \frac{k_1 K_{\mathrm{d;UIC2}}[\mathrm{ATP}]}{\left\{[\mathrm{UIC2}]+K_{\mathrm{d;UIC2}}\left(1+k_1 T_2\right)\right\}[\mathrm{ATP}]+\left([\mathrm{UIC2}]+K_{\mathrm{d;UIC2}}\right)K_{\mathrm{d;ATP}}} = \\ &= \frac{k_1 K_{\mathrm{d;UIC2}}}{[\mathrm{UIC2}]+K_{\mathrm{d;UIC2}}\left(1+k_1 T_2\right)} \cdot \frac{[\mathrm{ATP}]}{[\mathrm{ATP}]+K_{\mathrm{d;ATP}} \cdot \frac{[\mathrm{UIC2}]+K_{\mathrm{d;UIC2}}}{[\mathrm{UIC2}]+K_{\mathrm{d;UIC2}}\left(1+k_1 T_2\right)}} = \\ &= V_{\max} \cdot \frac{[\mathrm{ATP}]}{[\mathrm{ATP}]+K_{\mathrm{M}}}\end{aligned}$$

where

$$
\begin{aligned}
K_{\mathrm{M}} &= K_{\mathrm{d;ATP}} \cdot \frac{[\mathrm{UIC2}] + K_{\mathrm{d;UIC2}}}{[\mathrm{UIC2}] + K_{\mathrm{d;UIC2}}\left(1 + k_1 T_2\right)} = \\
&= \frac{K_{\mathrm{d;ATP}}}{\dfrac{[\mathrm{UIC2}] + K_{\mathrm{d;UIC2}}\left(1 + k_1 T_2\right)}{[\mathrm{UIC2}] + K_{\mathrm{d;UIC2}}}} = \\
&= \frac{K_{\mathrm{d;ATP}}}{1 + \dfrac{K_{\mathrm{d;UIC2}}}{[\mathrm{UIC2}] + K_{\mathrm{d;UIC2}}} \cdot k_1 T_2} = \\
&= \frac{K_{\mathrm{d;ATP}}}{1 + \dfrac{K_{\mathrm{d;UIC2}}}{[\mathrm{UIC2}] + K_{\mathrm{d;UIC2}}} \cdot \dfrac{T_2}{T_{1;\,\mathrm{min}}}}
\end{aligned}
\tag{1}
$$

and

$$
\begin{aligned}
V_{\mathrm{max}} &= \frac{k_1 K_{\mathrm{d;UIC2}}}{[\mathrm{UIC2}] + K_{\mathrm{d;UIC2}}\left(1 + k_1 T_2\right)} = \\
&= \frac{1}{\dfrac{[\mathrm{UIC2}]}{K_{\mathrm{d;UIC2}}} \cdot \dfrac{1}{k_1} + \dfrac{1}{k_1} + T_2} = \\
&= \frac{1}{\left(1 + \dfrac{[\mathrm{UIC2}]}{K_{\mathrm{d;UIC2}}}\right) \cdot T_{1;\mathrm{min}} + T_2}
\end{aligned}
\tag{2}
$$

2. Fractional labeling by UIC2

Let Y denote the fraction of Pgp protein labeled by UIC2 at steady state. Then Y is obtained as

$$Y = \frac{\left[X_0^*\right]+\left[X_1^*\right]}{\left[X_0^*\right]+\left[X_1^*\right]+\left[X_0\right]+\left[X_1\right]+\left[X_2\right]+...\left[X_\mathrm{n}\right]} =$$

$$= \frac{\left[X_0^*\right]+\left[X_1^*\right]}{\left[X_0^*\right]+\left[X_1^*\right]+\left[X_0\right]+\left[X_1\right]} \cdot \frac{\left[X_0^*\right]+\left[X_1^*\right]+\left[X_0\right]+\left[X_1\right]}{\left[X_0^*\right]+\left[X_1^*\right]+\left[X_0\right]+\left[X_1\right]+\left[X_2\right]+...\left[X_\mathrm{n}\right]} =$$

$$= \frac{[\mathrm{UIC2}]}{[\mathrm{UIC2}]+K_{\mathrm{d;UIC2}}} \cdot \frac{T_1}{T} =$$

$$= \frac{[\mathrm{UIC2}]}{[\mathrm{UIC2}]+K_{\mathrm{d;UIC2}}} \cdot \frac{\frac{[\mathrm{UIC2}]+K_{\mathrm{d;UIC2}}}{k_1 K_{\mathrm{d;UIC2}}} \cdot \frac{[\mathrm{ATP}]+K_{\mathrm{d;ATP}}}{[\mathrm{ATP}]}}{\frac{[\mathrm{UIC2}]+K_{\mathrm{d;UIC2}}}{k_1 K_{\mathrm{d;UIC2}}} \cdot \frac{[\mathrm{ATP}]+K_{\mathrm{d;ATP}}}{[\mathrm{ATP}]} + T_2} =$$

$$= \frac{[\mathrm{UIC2}]}{[\mathrm{UIC2}]+K_{\mathrm{d;UIC2}}} \cdot \frac{[\mathrm{ATP}]+K_{\mathrm{d;ATP}}}{[\mathrm{ATP}]+K_{\mathrm{d;ATP}} + \frac{K_{\mathrm{d;UIC2}}}{[\mathrm{UIC2}]+K_{\mathrm{d;UIC2}}} \cdot k_1 T_2 [\mathrm{ATP}]}$$

2.1. Expressing [ATP]-dependence of UIC2-labeling (at a fixed [UIC2]:

$$Y = \frac{[\mathrm{UIC2}]}{[\mathrm{UIC2}]+K_{\mathrm{d;UIC2}}} \cdot \frac{[\mathrm{ATP}]+K_{\mathrm{d;ATP}}}{[\mathrm{ATP}]+K_{\mathrm{d;ATP}} + \frac{K_{\mathrm{d;UIC2}}}{[\mathrm{UIC2}]+K_{\mathrm{d;UIC2}}} \cdot k_1 T_2 [\mathrm{ATP}]} =$$

$$= \frac{[\mathrm{UIC2}]}{[\mathrm{UIC2}]+K_{\mathrm{d;UIC2}}} \cdot \frac{[\mathrm{ATP}]+K_{\mathrm{d;ATP}}}{K_{\mathrm{d;ATP}} + \left(1+\frac{K_{\mathrm{d;UIC2}}}{[\mathrm{UIC2}]+K_{\mathrm{d;UIC2}}} \cdot k_1 T_2\right)[\mathrm{ATP}]} =$$

$$= \frac{\frac{[\mathrm{UIC2}]}{[\mathrm{UIC2}]+K_{\mathrm{d;UIC2}}} \cdot \frac{1}{1+\frac{K_{\mathrm{d;UIC2}}}{[\mathrm{UIC2}]+K_{\mathrm{d;UIC2}}} \cdot k_1 T_2} \cdot [\mathrm{ATP}] + \frac{[\mathrm{UIC2}]}{[\mathrm{UIC2}]+K_{\mathrm{d;UIC2}}} \cdot \frac{K_{\mathrm{d;ATP}}}{1+\frac{K_{\mathrm{d;UIC2}}}{[\mathrm{UIC2}]+K_{\mathrm{d;UIC2}}} \cdot k_1 T_2}}{[\mathrm{ATP}] + \frac{K_{\mathrm{d;ATP}}}{1+\frac{K_{\mathrm{d;UIC2}}}{[\mathrm{UIC2}]+K_{\mathrm{d;UIC2}}} \cdot k_1 T_2}} =$$

$$= \frac{Y_0 \cdot K_\mathrm{A} + Y_\infty \cdot [\mathrm{ATP}]}{[\mathrm{ATP}]+K_\mathrm{A}}$$

where

$$Y_0 = \frac{[\mathrm{UIC2}]}{[\mathrm{UIC2}] + K_{\mathrm{d;UIC2}}},$$

$$\begin{aligned} Y_\infty &= \frac{[\mathrm{UIC2}]}{[\mathrm{UIC2}] + K_{\mathrm{d;UIC2}}} \cdot \frac{1}{1 + \frac{K_{\mathrm{d;UIC2}}}{[\mathrm{UIC2}] + K_{\mathrm{d;UIC2}}} \cdot k_1 T_2} = \\ &= \frac{[\mathrm{UIC2}]}{[\mathrm{UIC2}] + K_{\mathrm{d;UIC2}}} \cdot \frac{1}{1 + \frac{K_{\mathrm{d;UIC2}}}{[\mathrm{UIC2}] + K_{\mathrm{d;UIC2}}} \cdot \frac{T_2}{T_{1;\min}}} \end{aligned},$$

and

$$\begin{aligned} K_{\mathrm{A}} &= \frac{K_{\mathrm{d;ATP}}}{1 + \frac{K_{\mathrm{d;UIC2}}}{[\mathrm{UIC2}] + K_{\mathrm{d;UIC2}}} \cdot k_1 T_2} = \\ &= \frac{K_{\mathrm{d;ATP}}}{1 + \frac{K_{\mathrm{d;UIC2}}}{[\mathrm{UIC2}] + K_{\mathrm{d;UIC2}}} \cdot \frac{T_2}{T_{1;\min}}} \end{aligned} \qquad (7)$$

Specifically,

$$\frac{Y_\infty}{Y_0} = \frac{1}{1 + \frac{K_{\mathrm{d;UIC2}}}{[\mathrm{UIC2}] + K_{\mathrm{d;UIC2}}} \cdot \frac{T_2}{T_{1;\min}}} \qquad (8)$$

2.2. Expressing [UIC2]-dependence of UIC2-labeling (at a fixed [ATP]):

$$Y = \frac{[\mathrm{UIC2}]}{[\mathrm{UIC2}] + K_{\mathrm{d;UIC2}}} \cdot \frac{[\mathrm{ATP}] + K_{\mathrm{d;ATP}}}{[\mathrm{ATP}] + K_{\mathrm{d;ATP}} + \frac{K_{\mathrm{d;UIC2}}}{[\mathrm{UIC2}] + K_{\mathrm{d;UIC2}}} \cdot k_1 T_2 [\mathrm{ATP}]} =$$

$$= \frac{[\mathrm{UIC2}]\left([\mathrm{ATP}] + K_{\mathrm{d;ATP}}\right)}{[\mathrm{UIC2}]\left([\mathrm{ATP}] + K_{\mathrm{d;ATP}}\right) + K_{\mathrm{d;UIC2}}\left([\mathrm{ATP}] + K_{\mathrm{d;ATP}}\right) + k_1 K_{\mathrm{d;UIC2}} T_2 [\mathrm{ATP}]} =$$

$$= \frac{[\mathrm{UIC2}]}{[\mathrm{UIC2}] + K_{\mathrm{d;UIC2}} + k_1 K_{\mathrm{d;UIC2}} T_2 \cdot \frac{[\mathrm{ATP}]}{[\mathrm{ATP}] + K_{\mathrm{d;ATP}}}} =$$

$$= \frac{[\mathrm{UIC2}]}{[\mathrm{UIC2}] + K_{\mathrm{d;UIC2}} \cdot \left(1 + k_1 T_2 \cdot \frac{[\mathrm{ATP}]}{[\mathrm{ATP}] + K_{\mathrm{d;ATP}}}\right)} =$$

$$= \frac{[\mathrm{UIC2}]}{[\mathrm{UIC2}] + K_{\mathrm{d;UIC2}} \cdot \left(1 + \frac{[\mathrm{ATP}]}{[\mathrm{ATP}] + K_{\mathrm{d;ATP}}} \cdot \frac{T_2}{T_{1;\mathrm{min}}}\right)} =$$

$$\frac{[\mathrm{UIC2}]}{[\mathrm{UIC2}] + K_{\mathrm{U}}} \quad ,$$

where

$$K_{\mathrm{U}} = K_{\mathrm{d;UIC2}} \cdot \left(1 + \frac{[\mathrm{ATP}]}{[\mathrm{ATP}] + K_{\mathrm{d;ATP}}} \cdot \frac{T_2}{T_{1;\mathrm{min}}}\right) \tag{9}$$

References for Supplementary Information

1 Sarkadi, B., Price, E. M., Boucher, R. C., Germann, U. A. & Scarborough, G. A. Expression of the human multidrug resistance cDNA in insect cells generates a high activity drug-stimulated membrane ATPase. J Biol Chem **267**, 4854-4858 (1992).

2 Mechetner, E. B. *et al.* P-glycoprotein function involves conformational transitions detectable by differential immunoreactivity. Proc Natl Acad Sci U S A **94**, 12908-12913 (1997).

3 Stein, W. D. Kinetics of the multidrug transporter (P-glycoprotein) and its reversal. Physiol Rev **77**, 545-590 (1997).

4 Al-Shawi, M. K., Polar, M. K., Omote, H. & Figler, R. A. Transition state analysis of the coupling of drug transport to ATP hydrolysis by P-glycoprotein. J Biol Chem **278**, 52629-52640 (2003).

5 Mechetner, E. B. & Roninson, I. B. Efficient inhibition of P-glycoprotein-mediated multidrug resistance with a monoclonal antibody. Proc Natl Acad Sci U S A **89**, 5824-5828 (1992).

6 Szaloki, G. *et al.* The strong in vivo anti-tumor effect of the UIC2 monoclonal antibody is the combined result of Pgp inhibition and antibody dependent cell-mediated cytotoxicity. PLoS One **9**, e107875 (2014).

7 Goda, K. *et al.* Complete inhibition of P-glycoprotein by simultaneous treatment with a distinct class of modulators and the UIC2 monoclonal antibody. J Pharmacol Exp Ther **320**, 81-88 (2007).

8 Csanady, L., Vergani, P. & Gadsby, D. C. Strict coupling between CFTR's catalytic cycle and gating of its Cl- ion pore revealed by distributions of open channel burst durations. Proc Natl Acad Sci U S A **107**, 1241-1246 (2010).
